# From target analysis to suspect and non-target screening of endocrine-disrupting compounds in human urine

**DOI:** 10.1007/s00216-022-04250-w

**Published:** 2022-07-29

**Authors:** Mikel Musatadi, Claudia Caballero, Leire Mijangos, Ailette Prieto, Maitane Olivares, Olatz Zuloaga

**Affiliations:** 1 Department of Analytical Chemistry, University of the Basque Country (UPV/EHU), Leioa, Basque Country 48940 Spain; 2 Research Centre for Experimental Marine Biology and Biotechnology (PiE), University of the Basque Country (UPV/EHU), Basque Country Plentzia, 48620 Spain

**Keywords:** Endocrine-disrupting compounds, Target analysis, Liquid chromatography tandem mass spectrometry, Suspect and non-target screening, High-resolution tandem mass spectrometry

## Abstract

In the present work, a target analysis method for simultaneously determining 24 diverse endocrine-disrupting compounds (EDCs) in urine (benzophenones, bisphenols, parabens, phthalates and antibacterials) was developed. The target analysis approach (including enzymatic hydrolysis, clean-up by solid-phase extraction and analysis by liquid chromatography coupled to tandem mass spectrometry (LC–MS/MS)) was optimized, validated and applied to volunteers’ samples, in which 67% of the target EDCs were quantified. For instance, benzophenone-3 (0.2–13 ng g^−1^), bisphenol A (7.7–13.7 ng g^−1^), methyl 3,5-dihydroxybenzoate (8–254 ng g^−1^), mono butyl phthalate (2–17 ng g^−1^) and triclosan (0.3–9 ng g^−1^) were found at the highest concentrations, but the presence of other analogues was detected as well. The developed target method was further extended to suspect and non-target screening (SNTS) by means of LC coupled to high-resolution MS/MS. First, well-defined workflows for SNTS were validated by applying the previously developed method to an extended list of compounds (83), and then, to the same real urine samples. From a list of approximately 4000 suspects, 33 were annotated at levels from 1 to 3, with food additives/ingredients and personal care products being the most abundant ones. In the non-target approach, the search was limited to molecules containing S, Cl and/or Br atoms, annotating 4 pharmaceuticals. The results from this study showed that the combination of the lower limits of detection of MS/MS and the identification power of high-resolution MS/MS is still compulsory for a more accurate definition of human exposome in urine samples.

**Graphical abstract:**

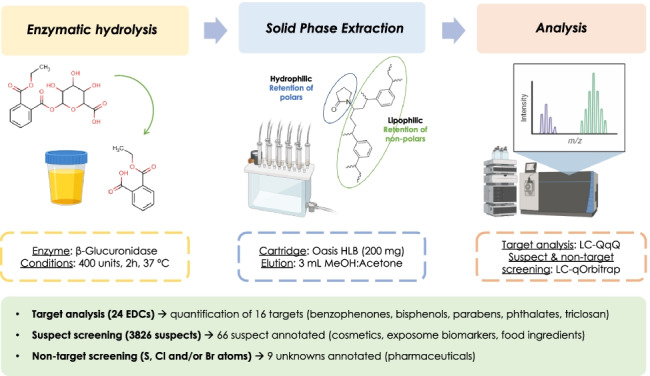

**Supplementary Information:**

The online version contains supplementary material available at 10.1007/s00216-022-04250-w.

## Introduction

The number of synthetic organic compounds produced and used nowadays is overwhelming [1]. Despite their utility, some of them pose a serious threat not only to the environment, but also to humans. In fact, around 90% of chronic human diseases can be linked to environmental factors, unlike the modest 10% that can be explained through genetics [2, 3]. From that environmental context, the concept of exposome emerged in 2005 [4]. Nowadays, the exposome engages all kind of exposures and factors that threaten humans throughout all our lifespans [5], and the quest for known and unknown chemical compounds is a key factor for its decoding.

In that framework, endocrine-disrupting compounds (EDCs) have been in the spotlight in the last decade since they can interfere with the endocrine system leading to, for instance, mutagenic, carcinogenic or hepatotoxic effects [6]. Some examples concerning EDCs include (i) bisphenols [7–9], (ii) benzophenones [10, 11], (iii) parabens [12–14], (iv) phthalates [15, 16] and (v) organochlorides such as triclosan (TCS) and triclocarban (TCC) [17–19].

Biomonitoring of those xenobiotics (i.e., compounds that do not occur naturally in the human organism [20]) as well as their respective phase I and phase II metabolites [21, 22] is often performed using urine samples, as it is shown in several epidemiological and clinic studies [8, 17, 23]. In order to quantify them, most target methodologies require a deconjugation reaction to transform phase II metabolites, mostly glucuronides and sulphate conjugates, into the unconjugated form [24, 25]. Although direct quantification of free and conjugated compounds could improve the interpretation of results in clinical research, a limited number of conjugate standards is available [26]. Afterwards, the unconjugated metabolites are extracted from the urine by a clean-up step, which is also used to remove interferences present in the matrix (endogenous compounds) and, therefore, to avoid signal suppression/enhancement (matrix effects) during their analytical determination. Solid-phase extraction (SPE) is the preferred technique when dealing with several EDC families [15, 25, 27], specially using reverse-phase (RP) polymeric sorbents [28] or mixed-mode SPE cartridges [29].

As for the analysis, liquid chromatography (LC) coupled to tandem mass spectrometry (MS/MS) is the most used technique to analyse organic compounds in urine [30, 31]. Low-resolution LC–MS/MS is used for target analysis and quantification of EDCs due to its low detection limits and robust performance [14, 32]. Within that targeted analysis context, most studies in the literature are focused on a reduced number of EDC families with similar chemical properties, instead of developing methods to simultaneously determine a wide variety of compounds with different chemical behaviours.

Nowadays, the emergence of high-resolution mass spectrometry (HRMS) has opened up new opportunities to look for xenobiotics or metabolites that are rarely followed in target analysis methods [33]. That way, suspect or non-target screening (SNTS) methods are progressively developing [34]. However, the mentioned approaches are major challenges in analytical chemistry, since the abundant and complex data obtained from high-resolution tandem mass spectrometry (HRMS/MS) makes the elucidation of unknowns a rough task [35, 36]. In that sense, extensive LC-HRMS/MS libraries are required [37, 38], as well as unambiguous criteria regarding quality control and quality assurance (QC/QA) [39] and identification confidence [40]. Moreover, sample preparation should achieve a balance between selectivity, by preserving as many compounds as possible, and sensitivity, by limiting matrix interferences [39].

Despite the mentioned difficulties, STNS methods are especially interesting to gather information for understanding and decoding the human exposome by finding relevant biomarkers. Therefore, the objectives of the present work have been, on the one hand, (i) to optimize and validate a target analysis method to simultaneously determine several EDC families (5 benzophenones, 6 bisphenols, 6 parabens, 5 phthalates and 2 antibacterial) in human urine by LC–MS/MS and, on the other hand, (ii) to extend the method to SNTS by LC-HRMS/MS.

## Materials and methods

### Reagents and solutions

In the target analysis method, 24 EDCs consisting of 6 bisphenols, 5 benzophenones, 6 parabens, 5 phthalate phase I metabolites and 2 antibacterials were included based on the literature, as well as 4 isotopically labelled standards. Moreover, 59 additional compounds were introduced in the experiments performed to extend the target analysis method to SNTS. These compounds consisted of exposome biomarkers, such as pharmaceuticals, industrial chemicals, perfluorinated alkyl substances (PFAS) and biocides containing either Cl, Br or S in their structure. All the information concerning the analytes and surrogates is compiled in Table S1 in the Supplementary information (SI). Moreover, the model compounds used in the Retention Time Indices Platform (RTI, http://rti.chem.uoa.gr/) are also presented in Table S1 in the SI.

As there are no real urine samples to be used as blanks, a synthetic urine was used for preparing blank and quality control (QC) samples for the optimization and validation of the methods (see Sect. 2.1 in SI) [41]. Information about the rest of the reagents and solutions used can also be found in Sect. 2.1 of SI.

### Development of the target method by UHPLC-ESI-QqQ

#### Sample treatment

Several variables affecting enzymatic hydrolysis and clean-up were evaluated by spiking synthetic urine with the target analytes in order to get 100 ng g^−1^ in the final extract. All the results were statistically evaluated using an analysis of variance (ANOVA) at a 95% confidence level.

Regarding enzymatic hydrolysis, β-glucuronidase enzyme units (400 and 4000, by adding 20 and 200 µL, respectively) and hydrolysis time (2 and 12 h) were studied to optimize deconjugation of glucuronides present in urine samples. To that end, 1 mL of synthetic urine was thawed to room temperature and spiked at 20 ng g^−1^ with bisphenol A glucuronide (BPA-G) alongside the rest of the xenobiotics except free bisphenol A (BPA). Two hundred microliters of ammonium acetate (NH_4_OAc, 1 M, pH 5.0) was added to ensure the proper media for β-glucuronidase activity, followed by the corresponding volume of β-glucuronidase solution. The deconjugation reaction was performed at 37 °C for optimum enzymatic activity. All experiments were carried out using three replicates (*n* = 3), and in all assays, the reaction was stopped by adding 2 mL of phosphate buffer (0.1 M, pH 2.0). The deconjugation efficiency was studied by following the percentage of BPA-G left after the hydrolysis, as well as the conversion to free BPA.

For the clean-up, three types of SPE cartridges were tested according to the literature [15, 17, 18, 25, 29]: (i) RP SPE, (ii) mixed-mode SPE combining RP with anion exchange and (iii) mixed-mode SPE combining RP with cation exchange. For RP SPE, polymeric-based Oasis HLB (6 mL, 200 mg, 30 µm, Waters, Milford, MA, USA) cartridges were used, while the following elution solvents were evaluated: (i) acetonitrile (AcN), (ii) ethyl acetate (EtOAc), (iii) methanol (MeOH) and several MeOH combinations such as (iv) MeOH:acetone, (v) MeOH:dichloromethane (DCM) and (vi) MeOH:EtOAc (all in 50:50, v/v). Moreover, 4 aliquots from 3 to 12 mL were recovered to study the elution profile using MeOH:acetone. Under optimal conditions, the cartridges were activated and equilibrated with 5 mL MeOH:acetone, 5 mL Milli-Q water and 5 mL phosphate buffer (0.1 M, pH 2.0). Then, the urine samples were loaded onto the cartridges and subsequently washed with 2 mL formic acid/formate (HCOOH/HCOO^−^) buffer (1 M, pH 2.0) and 5 mL Milli-Q water. Finally, the cartridges were fully dried under vacuum and the analytes were eluted with 3 mL MeOH:acetone (50:50, v/v).

Besides, Oasis MAX (6 mL, 150 mg, 30 µm, Waters) cartridges were selected for mixed-mode SPE combining RP and strong anion exchange, while TELOS neo PCX (3 mL, 100 mg, 50 µm, Kinesis) cartridges were used for mixed-mode SPE combining strong cation exchange with RP. A similar extraction procedure explained hereinafter was tested for both cases. First, the cartridges were activated and equilibrated with 5 mL of the following solvents: (i) EtOAc, (ii) MeOH, (iii) Milli-Q water and (iv) phosphate buffer (0.1 M, pH 2.0). After the urine samples were loaded, the cartridges were cleaned-up using 10 mL Milli-Q water and fully dried at vacuum. Lastly, the analytes were eluted with 12 mL MeOH followed by 12 mL EtOAc. In the case of the cationic exchanger, an additional 5 mL of MeOH containing 1% ammonia (NH_4_OH in solution) was used. The elution solvent nature and volume were not studied in the mixed-mode SPE.

After elution, 40 µL of dimethyl sulfoxide (DMSO) was added to the eluates as evaporation keeper and they were subsequently evaporated to 40 µL with a gentle stream of N_2_ at 35 °C using the Turbovap LC Evaporator (Zymark, Biotage, Uppsala, Sweden). Lastly, the extracts were diluted to 200 µL with HPLC water (H_2_O), filtered through polypropylene filters (0.22 µm, Phenomenex, Torrance, CA, USA) and kept in the freezer at – 20 °C in chromatography vials until analysis. Recovery and matrix effect were considered as quantitative criteria to compare the effectiveness of the tested cartridges (see Sect. 2.2.1 in SI for further details).

#### UHPLC-ESI–MS/MS analysis

The separation and detection of the target analytes were carried out by a UHPLC system (Agilent 1290 Infinity II) coupled to a triple quadrupole (QqQ) mass analyser (Agilent Technologies 6430 Triple Quad), equipped with a binary pump, a degasifying system, an automatic injector and an electrospray ionization (ESI) interface. The separation of the analytes was performed using an ACE UltraCore 2.5 SuperC18 (2.1 mm × 100 mm, 2.5 µm, Avantor, Symta, Madrid, Spain) chromatographic column that has a working pH range of 1.5–11, equipped with an UltraCore Super C18 UHPLC guard precolumn placed in an ACE UHPLC guard holder (both purchased from Avantor, Symta). The column temperature was maintained at 35 °C and 7 µL was selected as injection volume.

For the mobile phases, UHPLC water (A line) and MeOH (B line) were used at pH 2.5 (0.1% HCOOH) and 10.5 (0.05% NH_4_OH) to ensure ionization of all compounds. The flow rate was set to 0.3 mL min^−1^, and it was continuously in-line filtered through an ACE UltraCore 5 SuperC18 (2.1 mm × 30 mm, 5 µm, Avantor, Symta) column placed before the injector in order to reduce interfering compounds coming from the LC equipment. Further details on LC-QqQ analysis are included in Sect. 2.2.2 in SI.

#### Target method validation

The target analysis method by LC-QqQ was validated at three concentration levels (3 ng g^−1^, 6 ng g^−1^ and 30 ng g^−1^ in urine) in three consecutive days using 5 replicates of spiked synthetic urine samples (QC samples) [42]. Among the figures of merit of the validation (QC criteria), absolute and apparent recoveries (trueness), repeatability (intra-day precision), reproducibility (inter-day precision), instrumental and procedural limits of quantification (iLOQs and pLOQs, respectively) and parameters related to calibration curves’ linearity (upper limits and determination coefficients (*r*^2^)) were determined. The definition of the parameters is included in Sect. 2.2.3 in SI.

Regarding the quality assurance (QA) of the analytical sequence, all samples were randomly injected, while clean MeOH was introduced every 5 samples to check for possible carryover. 50 ng g^-1 ^calibration point was also injected throughout the sequence every 10 samples as instrumental QC sample to study signal intensity drifts and retention time (RT) shifts. For analyte quantification criteria, both *m/z* transitions should be present in the sample with an error of 30% in the ratio of their abundances and RT should be within ± 0.1 min of the pure standard.

### Suspect and non-target screening by HRMS

Although the sample treatment method was developed for the target analysis of 24 EDCs, the power of HRMS was tested to see whether the developed procedure could be widened to SNTS of different classes of xenobiotics in urine samples. Globally, 83 compounds were studied, 24 of which had been included during target method development. Analytes chosen for the SNTS also included a wide range of polarities with log *D* values at acidic pHs (loading value at the SPE protocol) ranging from − 0.8 to 6.7 (see Table S1 in SI).

#### UHPLC-HESI-HRMS/MS analysis

A Dionex Ultimate 3000 UHPLC (Thermo Fisher Scientific, MA, USA) coupled to a high-performance Q Exactive Focus Orbitrap (qOrbitrap, Thermo Fisher Scientific) mass analyser with a heated electrospray ionization source (HESI, Thermo Fisher Scientific) was used for the analysis of the EDCs. Same optimized chromatographic conditions (precolumn, column, mobile phases, temperature, injection volume, gradient and flow) mentioned in “UHPLC-ESI–MS/MS analysis” for LC-QqQ were used for LC-qOrbitrap as well. The only difference was the absence of the column used for filtering the mobile phase due to steric inconveniences in the loop. Regarding qOrbitrap operating conditions, they were set according to the experience of the research group without further optimisation [43] and are detailed in Sect. 2.3.1 in SI.

#### Data processing

Three different data-treatment approaches (target, suspect and non-target analyses) were used to treat the collected data by LC-qOrbitrap. The target analysis approach was used to calculate the iLOQs at pH 2.5 and 10.5. To that end, TraceFinder 5.1 (Thermo Fisher Scientific) software was used, which contained the RT, the exact mass and the characteristic fragment ions of the selected compounds (see Table S3 in the SI). A 0.1-min window was allowed in the RTs, a 70% fitting in the isotopic patterns and a 5-ppm error in the monoisotopic mass (MS1) and the most characteristic fragments (MS2). Since lower iLOQs were achieved with LC-QqQ, the concentration of the EDCs in the volunteers’ urine samples was not determined by LC-qOrbitrap, and thus, it was only used for SNTS.

Compound Discoverer 3.2 (Thermo Fisher Scientific) was used for SNTS. With regard to the peak picking criteria, only features with a minimum peak area of 10^6^ were considered when the RSD of the replicates (*n* = 5) of each sample was lower than 30% and the ratio with respect to synthetic urine blanks was higher than 10. Specific settings and parameters of the software regarding the peak selection have been already established and described by the research group [44]. Moreover, only features with a Lorentzian peak shape [45] were manually selected for further annotation.

**Fig. 1:**
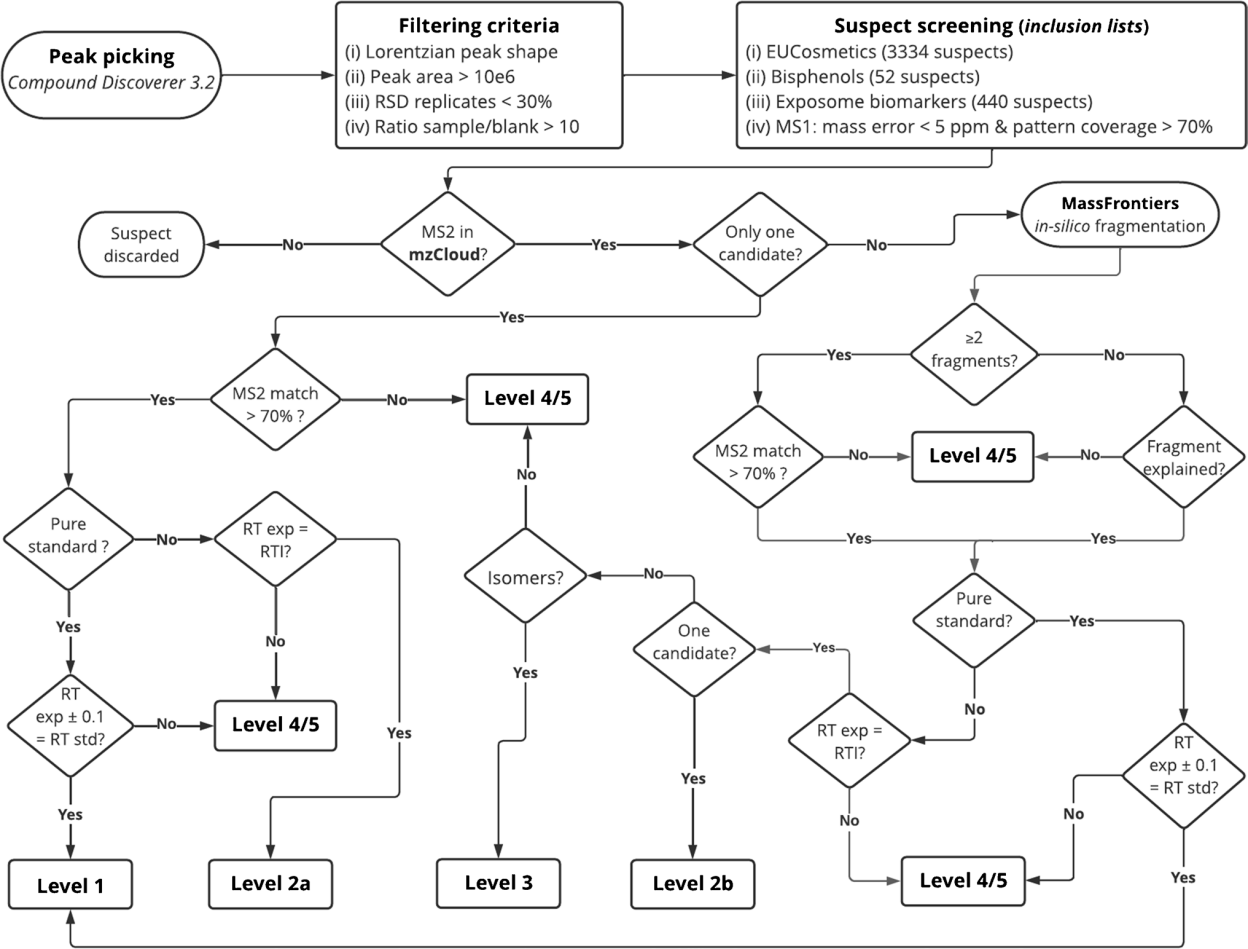
Scheme of the workflow used for the annotation of screened compounds in suspect screening

In the case of suspect screening (Fig. 1), three suspect lists obtained from the Norman network were used: (i) EUCosmetics [46], which is a combined inventory of ingredients employed in cosmetic products (SCCNFP/0389/00 Final) and revised inventory (Decision 2006/257/EC) with a total of 3334 suspects, (ii) a collection of 52 bisphenols [47] available at NILU from Table 3 of report 5/17 by KEMI (Swedish Chemicals Agency) and (iii) 440 exposome biomarkers [48] from Exposome-Explorer, which is a database dedicated to biomarkers of exposure to environmental risk factors for diseases. Therefore, the final suspect list contained a total of 3826 suspects, including the molecular formula, exact mass and the structure for each suspect. Besides, a list containing 1311 endogenous urine metabolites was obtained from the Human Metabolome Database (HMDB) to avoid false identification of endogenous compounds as xenobiotics [49]. A detailed description of the peak annotation workflow is included in Sect. 2.3.2 in SI.

Besides suspect screening, non-target screening was carried out limiting it to molecules containing Cl, Br and/or S due to the specific isotopic profiles of molecules containing those atoms [34]. When according to the isotopic pattern (SFit > 50 and pattern coverage > 90) a feature contained at least one atom of Cl, Br and/or S, candidates in the ChemSpider searched by Compound Discoverer 3.2 were considered. When available, mzCloud spectra were examined and, when not, in silico fragmentation was performed. Finally, RT was considered in the same terms as in the suspect screening workflow, as well as the annotation confidence levels (Fig. 1).

#### Quality control/quality assurance

Even though the concept of method validation is only used for target analysis methods, some QC/QA measures can be implemented for STNS as well [39]. A 10-point external calibration was prepared in the 0.5–200-ng g^−1^ range in H_2_O:DMSO (80:20, v/v) for calculating instrumental limits of identification (iLOIs) of the 83 compounds and, therefore, instrumental false negatives. In the case of the iLOIs, they were set as the lowest concentration level that could be unequivocally identified [50], meaning a mass error lower than 5 ppm, an isotopic profile and fragmentation spectra fit of at least 70% and a ± 0.1 min error in the RT.

Furthermore, 1-mL synthetic urine samples (*n* = 5) were fortified with the 83 compounds at 6 ng g^−1^ (QC samples), processed following the SPE method by Oasis HLB and analysed by UHPLC-qOrbitrap. Then, SNTS workflows (see “Data processing”) were used for identifying the analytes and evaluate losses. Procedural blanks (*n* = 5) were also processed to avoid identification of compounds coming from elsewhere. These additional experiments were only made with screening and annotation purposes, while quantitative analysis was out of the scope. Regarding the analytical sequence, the same QA criteria as in the target method validation were followed.

### Analysis of real urine samples

To test the applicability of the developed methods, real urine samples provided by 4 volunteers from the research group were analysed, while the obtention of environmental or epidemiological results was out of the scope of this work. Those samples were manipulated according to the indications of the Ethics Commission for Research and Teaching of the University of the Basque Country (CEISH-UPV/EHU, BOPV 32, 17/2/2014 M10 2021 124 and CEIAB-UPV/EHU, BOPV 32, 14/2/14, M30 2021 158). Sample handling is further explained in Sect. 2.4 in SI.

## Results and discussion

### Optimization of the target method by LC-QqQ

#### Enzymatic hydrolysis

The percentage of BPA-G left in all experiments after hydrolysis was lower than 5%, showing that the enzyme quantitatively deconjugated the glucuronide regardless of the reaction time (2 or 12 h) and enzyme units (400 or 4000) used. Moreover, average recoveries (*n* = 3) obtained for the rest of the compounds were statistically comparable (*p* > 0.05 for a 95% confidence level) showing no degradation at 37 °C independently of the reaction time (data not shown). Specifically, the recovery of BPA (all deconjugated from BPA-G) was 70%, the same as in the experiments spiking with BPA instead of BPA-G. Therefore, the loss observed could be attributed to the other steps in the procedure and not to the enzymatic hydrolysis. Bearing in mind all of the mentioned, the reaction was carried out overnight (12 h) during validation using 400 units of the enzyme (20 µL) for practical purposes in order to guarantee method throughput. Nevertheless, considering that the hydrolysis may differ for other glucuronide metabolites in urine, further investigation is required in that aspect studying other glucuronides [51]. Additionally, the hydrolysis of sulphate conjugates using β-glucuronidase/arylsulfatase enzymes should be addressed in future works as well.

#### Optimization of the clean-up step

As previously mentioned, three SPE approaches were tested during the clean-up step: (i) a RP approach using Oasis-HLB cartridges, (ii) a mixed-mode anionic exchanger approach using Oasis-MAX cartridges and (iii) a mixed-mode cationic exchanger using TELOS neo-PCX cartridges. Recoveries and matrix effects obtained with each solvent using Oasis HLB cartridges are presented in Figure S1 in the SI. As it can be observed in Figure S1a, the best elution solvents for Oasis HLB in terms of recoveries consisted in those containing MeOH, which could disrupt the polar-polar interaction between the target compounds and the polar groups in the Oasis HLB cartridge by hydrogen bonding [28]. In fact, analytes such as BP2, BPS, MDHB or TCC that contain –OH or –NH groups were not recovered in the absence of MeOH.

With the aim of incrementing the recoveries of analytes with less polarity, MeOH was combined with other more non-polar solvents (DCM, acetone and EtOAc). MeOH:DCM mixture rendered SPE recoveries higher than 100%, but the extracts obtained suffered from a stronger signal suppression at detection (Figure S1b). Pure MeOH, MeOH:acetone and MeOH:EtOAc provided better results regarding matrix effect. Finally, the MeOH:acetone mixture was chosen between the three solvents since it rendered recoveries closer to 100% for less polar analytes such as BP3 or BPZ, while the matrix effect was comparable as it can be seen in Figure S1b.

**Fig. 2:**
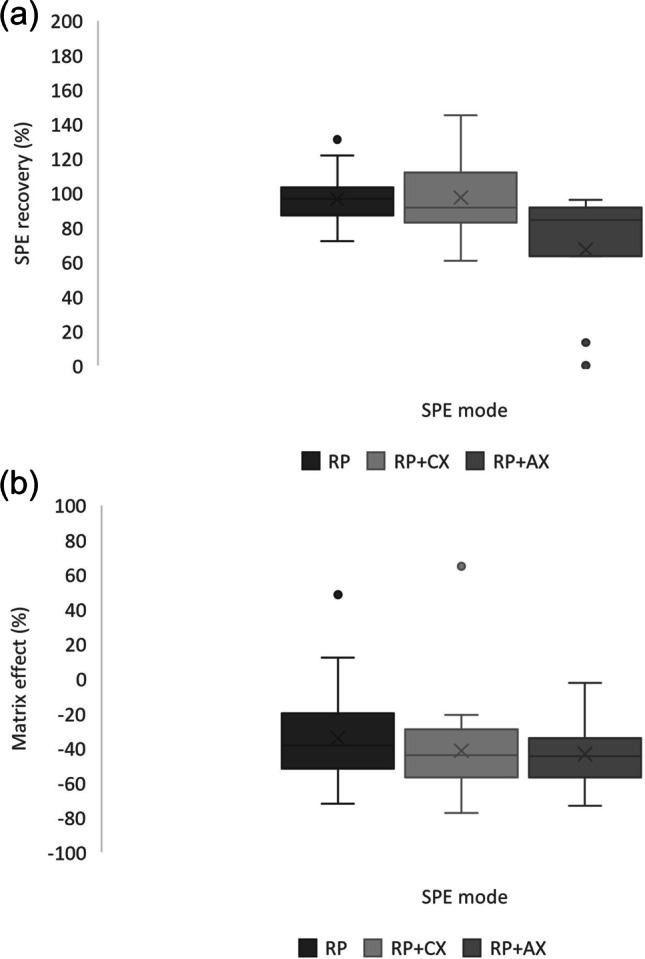
Boxplots of the **a** SPE recoveries and **b** matrix effects at detection obtained for the reverse phase (RP) and mixed mode using RP and cationic exchanger (CX) and RP and anionic exchanger (AX) cartridges by LC-QqQ

Even under optimum conditions, most target compounds suffered from signal suppression (20–30%) using Oasis HLB cartridges that could be due to the high salt content of urine despite the clean-up step. Therefore, mixed-mode cationic and anionic exchangers were tested aiming to decrease the concentration of salts in the final extract. In this sense, the objective would be the retention of cations (urea, creatinine, Na^+^) or anions (Cl^−^, PO_4_^3−^) present at high concentrations in urine using the ionic exchange mechanism, while the target compounds would be retained through the RP mechanism. The recoveries and matrix effects obtained for spiked synthetic urine are summarized in Fig. 2. Since the consecutive elution with EtOAc in the anionic exchanger and with EtOAc and MeOH (1% NH_4_OH) in the cationic exchanger did not increase the recoveries, that data is not shown. Moreover, results for Oasis HLB using MeOH:acetone 50:50 (v/v) as elution solvent are also included in Fig. 2 for a better comparison of the studied protocols.

As can be seen in Fig. 2a, TELOS neo PCX cartridges (RP + CX) rendered the highest recoveries for the mixed mode, statistically equal (*p* value > 0.05, 95% confidence level) to those obtained with Oasis HLB using MeOH:acetone as elution solvent. In terms of matrix effect (Fig. 2b), mixed-mode approaches showed a similar signal suppression comparable to Oasis HLB. Since the use of mixed-mode cartridges did not minimize signal suppression, Oasis HLB cartridges were chosen considering the ease to run the procedure. Once the SPE cartridge and solvent nature were fixed, an elution profile was performed by collecting four volumes of MeOH:acetone (50:50, v/v) from 3 to 12 mL. Based on the results (Figure S2 in the SI), 3 mL was sufficient for a quantitative elution of the target compounds.

### Target method validation: figures of merit

#### Instrumental limits of quantification and calibration ranges

**Fig. 3:**
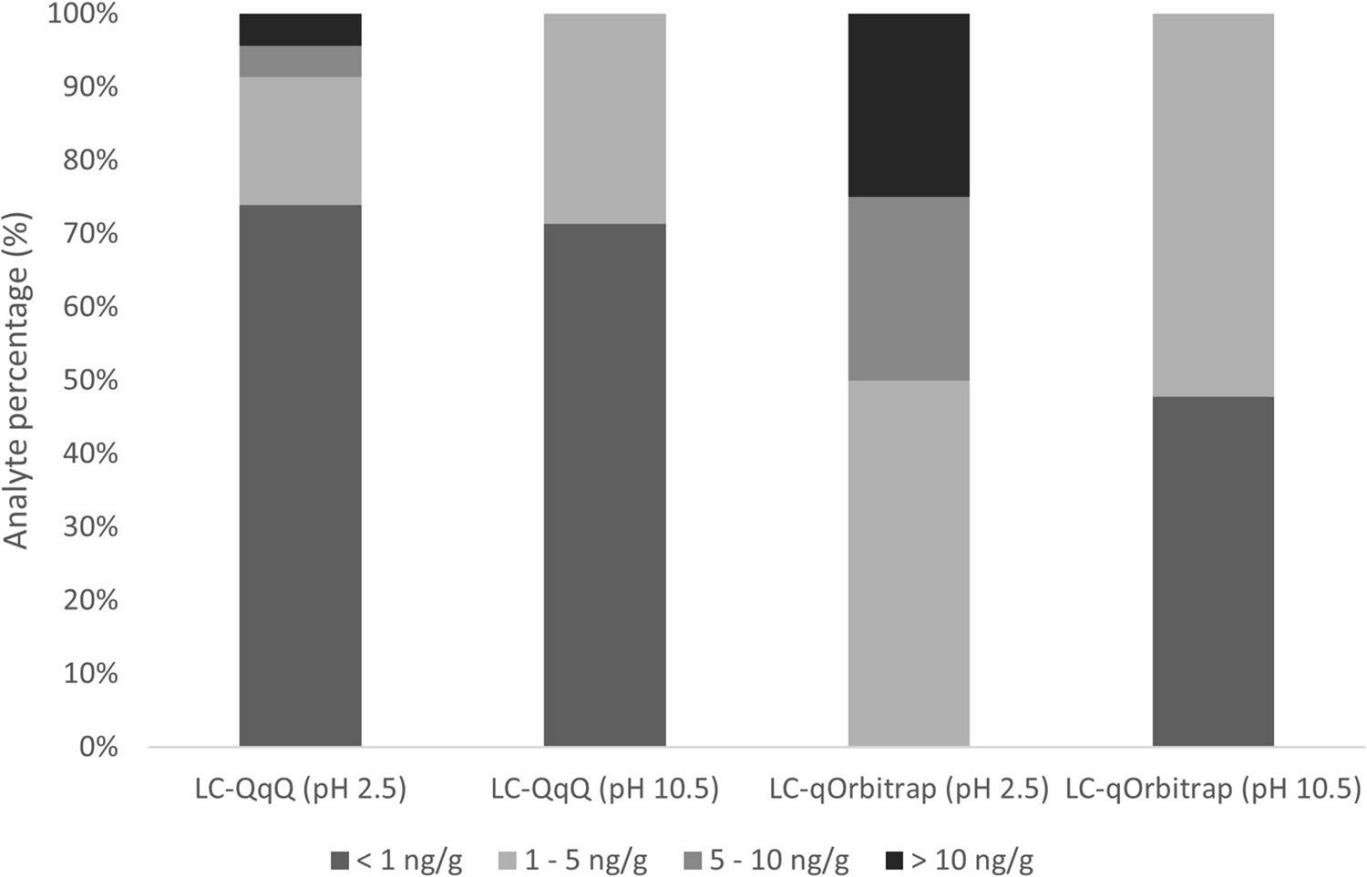
Instrumental limits of quantification obtained at pH 2.5 and 10.5 for LC-QqQ and LC-qOrbitrap

As it can be seen in Fig. 3, lower iLOQs were obtained with the LC-QqQ than with LC-qOrbitrap mass analyser, showing higher sensitivity of the DMRM mode than the Full MS-ddMS2. In fact, more than 70% of the analytes provided iLOQ values lower than 1 ng g^−1^ in the DMRM regardless of the pH of the mobile phase used. The case of the parabens is the most remarkable one in that sense, where a 10-time difference is appreciated comparing both detectors. In LC-QqQ in general, better values were achieved for the bisphenols in the basic pH, while parabens and phthalates showed lower iLOQ in the acidic media. On the contrary, most iLOQ values were between 1 and 5 ng g^−1^ for qOrbitrap, being more sensitive basic pH, especially for bisphenols, whose iLOQs were not included in the acidic pH for qOrbitrap. All iLOQs are collected in Table S4 in the SI.

All in all, the values obtained are comparable to those achieved in other works in the literature, which range between 0.5 and 2 ng g^−1^ [24, 52–54]. However, and as a tendency in analytical chemistry, in most of the works the LOQs are calculated using signal-to-noise (S/N) ratios. Compared to the S/N criteria for calculating iLOQs, the criteria applied in the present work are stricter since they take into account not only that the signal differs from the blank but also the fitting with the calibration curve and the precision [55].

Besides, upper limits of calibration ranges are also included in Table S4 for both LC-QqQ and LC-qOrbitrap at both pHs. Similar linearity ranges were observed for both detectors, showing in a slight tendency to curve at concentrations above 150–200 ng g^−1^ that fitted to quadratic calibration curves. However, that tendency was less evident in the case of LC-QqQ, in which also better determination coefficients (*r*^2^) were obtained. However, since those high concentrations are not expected in urine, most curves were limited to 100–150 ng g^−1^ in order to have linear curves and avoid quadratic fitting. Taking all into account, LC-QqQ was used for quantitative target analysis in real samples, while LC-qOrbitrap was limited to SNTS.

#### Trueness, precision and procedural limits of quantification

The individual absolute and apparent recoveries obtained are included in Tables S5 in SI, for each spiking level (3, 6 and 30 ng·g^−1^) at the three consecutive days, with their respective RSDs. The surrogate used for correction for each analyte is also included at each concentration level. In the cases where an analyte was ionized at both pHs, the one providing better iLOQs was considered (see Table S4).

**Fig. 4:**
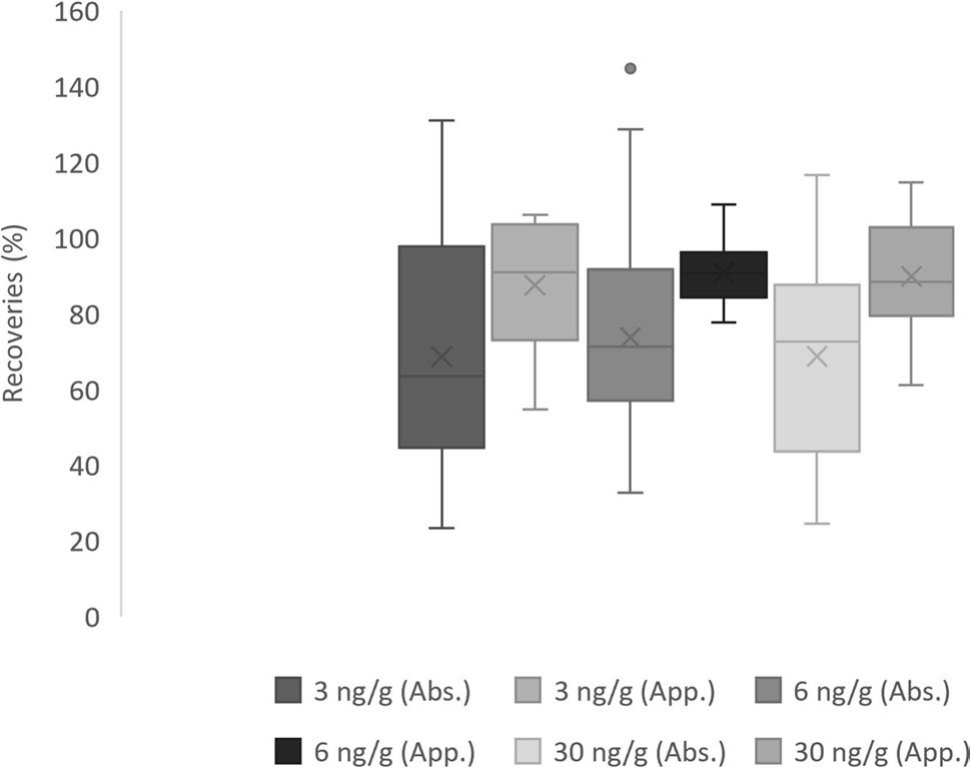
Boxplots of the average (*n* = 15) absolute (Abs.) and apparent (App.) recoveries obtained for synthetic urine spiked at low (3 ng g^−1^), medium (6 ng g^−1^) and high (30 ng g^−1^) concentration levels

Three compounds (BPP, TCC and TCS) could not be quantified at the lowest spiking level (3 ng g^−1^). For the rest of the compounds, absolute recoveries between 20 and 130% were obtained. At medium (6 ng g^−1^) and high (30 ng g^−1^) spiking levels, slightly better absolute recoveries were achieved (30–110%). According to the ANOVA, the absolute recoveries obtained were comparable within the different days at each spiking level (*F*_critic_ = 3.15 > *F*_calculated_ = 0.13, *F*_critic_ = 3.13 > *F*_calculated_ = 0.09 and *F*_critic_ = 3.13 > *F*_calculated_ = 0.31, for low, medium and high levels, respectively). Moreover, due to the proper surrogate correction, the apparent recoveries were also statistically comparable within the days (*F*_critic_ = 3.22 > *F*_calculated_ = 0.85, *F*_critic_ = 3.19 > *F*_calculated_ = 0.78 and *F*_critic_ = 3.21 > *F*_calculated_ = 0.98) since they ranged between 70 and 100%. Taking into consideration the ANOVA results shown, the method reproducibility (inter-day precision) throughout the days could be concluded. That outcome is crucial in exposome biomonitorization experiments, in which large sample sets need to be analysed so the division of them in several days is assured. Therefore, the average absolute and apparent recoveries obtained for the 3 days are shown for each level in Fig. 4. As can be seen in Fig. 4, the absolute and apparent recoveries are similar at the three concentration levels, which can be further confirmed by ANOVA (*F*_critic_ = 3.14 > *F*_calculated_ = 0.18 and *F*_critic_ = 3.2 > *F*_calculated_ = 0.2 for absolute and apparent recoveries, respectively).

Regarding the intra-day precision of the method (repeatability), the recoveries of the different days in each level were combined (*n* = 15) due to the mentioned method reproducibility, and RSD values of the absolute recoveries were not statistically comparable among spiking levels at a 95% confidence level according to ANOVA (*F*_critic_ = 3.13 < *F*_calculated_ = 8.45). That outcome could be explained by the higher RSD values obtained at the low spiking level, although only MDHB exceeded the 35% limit. For the rest of the analytes, most values were around 20–30% at the low spiking level, while lower RSDs (around 10–15%) were obtained at medium and high levels. Nevertheless, after surrogate correction, the RSDs of the apparent recoveries were comparable (*F*_critic_ = 3.21 > *F*_calculated_ = 3.01) since most ranged between 10 and 15%. Therefore, the method was considered repeatable.

In the literature, equivalent trueness and precision values have been obtained for this type of EDCs in urine using Oasis HLB. Nevertheless, most works have been limited to one or few families, such as phthalate metabolites [53], phthalates and BPA [56] or BPA and triclosan [57]. Despite surrogate correction, it would be interesting to assess potential losses in the filtration step, as well as the evaluation of the matrix effect at several concentration levels.

Lastly, the pLOQ values (see Table S4 for individual values) obtained were in the same order as the concentrations expected or detected in other works in urine [41, 52, 58, 59]. Regarding the pLOQs of LC-QqQ, most values obtained were below 0.5 ng g^−1^, while higher values were obtained for LC-qOrbitrap, ranging around 1–2 ng g^−1^.

### Quality control/quality assurance of suspect and non-target screening

#### Instrumental limits of identification

First, iLOIs were estimated for the 83 analytes using the workflows described in “Data processing,” and the results obtained are included in Table S3 alongside the MS2 match using mzCloud or in silico fragmentation. Due to the stricter requirements, iLOI values turned out to be higher that their respective iLOQ for the target analytes, since 90% of the analytes were ranging between 0.5 and 28.1 ng g^−1^. Considering all the 83 analytes, better values were achieved in the acidic pH in general. In fact, the iLOIs at pH 2.5 were mostly between 0.1 and 10.8 ng g^−1^, while at pH 10.5, they ranged between 1 and 22 ng g^−1^, due to the poorer fragmentation observed in the negative ionization mode rendering a lower identification power. Regarding the analytes that provided high iLOQs, MBuP, MEHHP, MEOHP, caffeine, acetaminophen, MBnP and 2-hydroxybenzothiazole had values higher than 50 ng g^−1^.

Most xenobiotics were screened using mzCloud, allowing the calculation of their respective iLOI. In the cases of BnP, MBnP, MEOHP, MEHHP and MDHB, however, they were positively identified using in silico fragmentation. In the cases of BP3 and BuP, the identification was done by either mzCloud or in silico match depending on the ionization mode. While BuP was identified by mzCloud match in the negative mode, it was positively annotated only by in silico fragmentation in the positive mode. The opposite occurred for BP3 as it can be observed in Figures S3a and S3b in SI for positive and negative modes, respectively. This could be explained because only fragmentation spectra in the positive and the negative mode, respectively, are available in mzCloud. It could be therefore highlighted that in silico fragmentation is a complementary tool for the annotation of non-targets. Finally, MeP and MBnP could only be identified in one of the ionization modes, negative mode for the first and positive for the latter, although they could be quantified in both ionization modes in LC-QqQ.

Nevertheless, iLOIs could not be calculated for all compounds due to different problems that would lead to instrumental false negatives. On the one hand, the fragmentation spectra obtained for some pure standards could not be explained either using mzCloud library or in silico fragmentation. This was the case of TCS, BPZ and BPP. Regarding TCS, the spectra in mzCloud contained the fragments 160.95664 Da and 141.98271 Da, while we only obtained a single fragment at 91.62964 Da (see Figure S4 in SI). In the cases of BPZ and BPP, those two compounds are not included in mzCloud and the in silico fragmentation was not able to explain the fragments obtained. On the other hand, some compounds were missed due to the filter of mzCloud match (> 70%), as occurred for cotinine (42%), chlortoluron (42%) benzothiazole (41%), mecoprop (36%) and fenthion (50%). It should be highlighted that except for cotinine, benzothiazole and fenthion, the rest of the compounds mentioned were ionized in the negative mode, which usually renders a poorer fragmentation spectrum as stated. Therefore, in-house libraries should be implemented not to miss such compounds.

In summary, in the case of pure standards, only 9.6% of the compounds (8 out of 83) could be considered false negatives using the established criteria, while 12.5% of the analytes included in the target method were not screened (3 out of 24). As for the calculation of iLOIs in human matrices, it is worth mentioning that there is a huge gap in the literature in that area [43], since LOIs have been mainly calculated in environmental matrices. The absence of LOIs in human matrices confirms the lack of harmonization in QC/QA measurements in STNS, as it has been previously stated by other experts in the field [39].

#### Screening of targets in quality control samples

After the application of the SNTS workflows to pure standards, spiked synthetic urine QC samples were evaluated to assess potential losses of the SPE protocol and matrix effect. In the case of BP8, although pure standards could be positively identified both in the positive and negative ionization modes, in spiked urine samples, fragmentation spectra were only obtained in the negative mode. In addition, no fragmentation spectra were obtained for MBnP, EDHB, 4-PBZ, 4,4′-DMA-BP, fenthion, TCC, oryzalin, quinmerac, ethion and PFOSA. Lastly, in the case of BPS, metribuzin and imidacloprid, although a fragmentation spectrum was obtained, the identification match was lower than 70% compared to the mzCloud library.

As a result, 13 additional analytes could not be satisfactorily screened due to the lack of extraction during SPE, potential losses in the evaporation or filtration step and/or matrix effect during the detection. Therefore, false negatives increased from a 9.6% in pure standards to a 22.9% in synthetic urine. Considering that 6 of 19 compounds that could not be identified using SNTS approaches had been previously used during method development, it could be highlighted that 78.0% of the compounds not considered during method development were satisfactorily identified, and consequently, that the method developed by Oasis HLB could be used for SNTS. Nevertheless, it should also be pinpointed that 6 of 24 targets (25.0%) used during method development could not be identified in a SNTS approach using LC-qOrbitrap in the FullMS-ddMS2 acquisition mode in synthetic urine, while they could be quantified even at lower concentration with a LC-QqQ low-resolution mass analyser in the DMRM mode. The mentioned drawbacks should be carefully assessed in future works.

### Application to real samples

#### Target analysis

The validated target analysis method by LC-QqQ was applied to the urine samples of four volunteers named A, B, C and D to test method viability. The average concentrations (*n* = 5, ng g^−1^ in wet weight) and the confidence intervals at 95% (defined as 2 s, where s corresponds to the standard deviation) measured by LC-QqQ are included in Table 1. Only those compounds above the pLOQ with a RSD lower than 35% are included. It should be stated that creatinine correction is discouraged for providing pollutants’ concentrations in urine [60], and therefore, it was not performed.

**Table 1:** Average (*n* = 5) concentrations (± 2 s, ng g^−1^) of the target EDCs in the volunteers’ samples treated by SPE-LC-QqQ, as well as concentrations found in the literature

EDC family	Analyte	This work				Literature		
		**A sample**	**B sample**	**C sample**	**D sample**	**Concentration (ng/mL)**	**Method**	**Reference**
***Benzophenones***	4OH-BP	0.7 ± 0.2	0.3 ± 0.2	0.2 ± 0.1	0.10 ± 0.06	0.11–0.24	SPE-LC–MS/MS	[61]
	BP1	3 ± 1	5.2 ± 0.5	< pLOQ	< pLOQ	1.38–5.64 0.7–2.7 < 234	SPE-LC–MS/MS SPE-LC–MS/MS ID-LC–MS/MS	[61] [59] [58]
	BP2	0.21 ± 0.04	0.4 ± 0.2	0.18 ± 0.06	< pLOQ	0.04–0.11 < 149	SPE-LC–MS/MS ID-LC–MS/MS	[61] [58]
	BP3	12 ± 1	9.1 ± 0.2	0.7 ± 0.2	0.7 ± 0.5	3.24–12.21 1.0–4.6 < 803	SPE-LC–MS/MS SPE-LC–MS/MS ID-LC–MS/MS	[61] [59] [58]
***Bisphenols***	BPA	13.6 ± 0.1	8.3 ± 0.6	< pLOQ	< pLOQ	0.3–0.9 < 30.7 1.7–44.8 10.8–88.5 0.52^a^, 1.15^b^ 0.2–12	SPE-LC–MS/MS ID-LC–MS/MS SPE-LC–MS/MS LLE-LC–MS/MS ID-LC–MS/MS DS-LC–MS/MS	[59] [58] [62] [63] [64] [32]
	BPAF	0.02 ± 0.01	< pLOQ	0.02 ± 0.01	< pLOQ	< LOQ 0.99–38.6 0.5–39	SPE-LC–MS/MS LLE-LC–MS/MS DS-LC–MS/MS	[59] [63] [32]
	BPS	0.6 ± 0.1	2.0 ± 0.6	1.3 ± 0.2	3 ± 2	0.1–0.2 0.15–2.45 1.35^a^, 2.02^b^ 0.5–8.5	SPE-LC–MS/MS LLE-LC–MS/MS ID-LC–MS/MS DS-LC–MS/MS	[59] [63] [64] [32]
***Parabens***	EDHB	1.3 ± 0.1	2.2 ± 0.6	1.3 ± 0.1	2.2 ± 0.8	–^c^	–^c^	–^c^
	EtP	0.2 ± 0.1	< pLOQ	< pLOQ	< pLOQ	4.2–48.9 < 273 4.66–2990 0.2–2300	SPE-LC–MS/MS ID-LC–MS/MS LLE-LC–MS/MS DS-LC–MS/MS	[59] [58] [63] [32]
	MDHB	241 ± 13	21 ± 6	34 ± 6	12 ± 4	–^c^	–^c^	–^c^
***Phthalates***	MBnP	0.48 ± 0.03	0.16 ± 0.03	0.18 ± 0.04	0.14 ± 0.01	0.2–1.0 0.30–2.33 8.44^a^, 16.97^b^	SPE-LC–MS/MS SPE-LC–MS/MS ID-LC–MS/MS	[59] [65] [64]
	MBuP	12 ± 5	4 ± 2	5 ± 3	10 ± 6	0.02–28.60	SPE-LC–MS/MS	[65]
	MEHHP	0.7 ± 0.2	0.5 ± 0.2	0.9 ± 0.4	1.12 ± 0.09	1.2–106 2.03–19.80	SPE-LC–MS/MS SPE-LC–MS/MS	[62] [65]
	MEHP	0.8 ± 0.1	0.7 ± 0.2	0.9 ± 0.2	0.7 ± 0.3	< 1.4 2.7–41.1 4.03–12.40	SPE-LC–MS/MS SPE-LC–MS/MS SPE-LC–MS/MS	[59] [62] [65]
	MEOHP	0.31 ± 0.09	0.31 ± 0.07	0.39 ± 0.08	0.69 ± 0.09	0.7–2.1 0.86–105 1.74–5.77	SPE-LC–MS/MS SPE-LC–MS/MS SPE-LC–MS/MS	[59] [62] [65]
***Antibacterials***	TCS	8 ± 1	0.4 ± 0.1	0.8 ± 0.5	< pLOQ	0.1–0.9 6.2–20.1 10.7–26.9	SPE-LC–MS/MS SPE-LC–MS/MS SPE-LC–MS/MS	[59] [66] [67]

^a^Geometric mean

^b^Mean

^c^Not found in the literature

In general, the highest concentrations of the target compounds were measured in sample A. Among benzophenones, BP3 was found at the highest concentrations in all samples followed by BP1 and BP2. The metabolite of benzophenone 4OH-BP was also detected in all the samples. In the case of bisphenols, although BPA was found at the highest concentration in samples A and B, BPS was quantified at lower concentrations in the four samples, indicating the introduction of alternatives of BPA in the market [68]. BPAF could also be quantified in samples A and C. Among parabens, the highest concentrations were found for MDHB (12–241 ng g^−1^), whereas EDHB and EtP were found at lower concentrations, showing the exposure to chemicals present in personal care products [58]. Regarding the phthalates, MBuP was the metabolite found by far at the highest concentration (4–12 ng g^−1^), while MBnP, MEHHP, MEHP and MEOHP were found at a similar concentration level, showing the exposure to plasticizers [65]. Finally, TCS was found at one order of magnitude higher in sample A than in the rest of the samples, comparable to the rest of EDCs quantified.

All in all, similar concentration levels of the EDCs were detected in this work comparing to the other works in the literature, with the exception of punctual high concentrations determined in some cases (see Table 1). It should be mentioned, however, that most works were limited to certain EDC families. Although these concentrations were at low ng g^-1^ levels, their effects should be further studied, especially considering the “cocktail effect” which could increase the toxicity of the chemicals when they are present together [69]. Finally, the presence of diverse EDCs from the same family suggests the usage of analogues of the already banned or regulated compounds. That outcome further increases the necessity of new laws and regulations.

#### Suspect and non-target screening

SNTS was also applied to the urine samples of the four healthy volunteers. The aim of obtaining epidemiological/environmental conclusions is far beyond the purpose of the experiments carried out, which was focused in testing the power of the developed method to identify unknown compounds using a SNTS approach. Table S6 in the SI includes the features annotated at levels 1–5 with both approaches.

Using the suspect workflow, a total of 66 features could be identified at levels 1–5. While 50% of the total features was annotated at levels 1–3, 30.3% and 19.7% were annotated at levels 4 and 5, respectively. Nine compounds could be identified at level 1, including acetaminophen (pharmaceutical), cotinine (nicotine metabolite), caffeine (stimulant), daidzein (food natural ingredient), genistein (food natural ingredient/personal care product), BP3 (UV filter), BPA (plasticizer), MBuP (plasticizer) and MEHP (plasticizer). Compared to target analysis, we missed 12 of the 16 compounds determined. In the case of 4OH-BP, BP1, MDHB and MEOHP, we could detect the peak, but no fragmentation spectrum was available, and identification was, therefore, not viable. In the case of BPS, BPAF, EDHB, EtP, MBnP, MEHHP and TCS, the signal was not intense enough and we could not even detect the peak. Finally, in the case of MDHB, we could detect the chromatographic peak but the quality of the MS1 was not good enough to deduce the molecular formula.

Among the 8 compounds annotated at level 2a, it could be highlighted that 4 of them corresponded to food additives, but 2 cosmetics, a household product and a drug product were annotated as well. In the case of level 2b, food additives, cosmetics and synthetic polymers could be screened. Regarding level 3, most suspects corresponded to food additives or stimulants. Due to the nature of the compounds identified using the suspect workflow, most of the compounds annotated at levels 1–3 were observed in most of the samples. However, differences could be observed according to the principal component analysis (PCA) run using the statistical package for multivariate data analysis available in Compound Discoverer 3.2 software (Figures S5). The first three principal components (PCs), PC1 (36.2%), PC2 (24.5%) and PC3 (21.0%), explained up to 81.7% of the whole variance in the case of the suspects annotated in the positive ionization mode. As can be observed in the PC2 vs PC1 scores plot diagram (see Figure S5a) for the positive ionization mode, sample C differed significantly from the blank and the rest of the samples analysed. Features #1, 2, 4, 16, 23, 24, 26, 28 and 29 (see Table S6) were the most significant in volunteer C according to the loadings plot (Figure S5b), which included acetaminophen, cotinine and, mostly, different food additives. On the contrary, samples D and B could be considered the closest to the blank with the least number of features detected. The PC3 vs PC1 score plot did not render further information and was not included. Furthermore, the negative ionization mode was not considered for PCA since only 5 compounds were annotated.

Besides, the non-target approach allowed the detection of 9 features annotated at levels 1–5: 1 at level 2a, 2 at level 2b, 1 at level 3 and 5 at level 5 (Table S6). Among the compounds annotated at level 2b, they corresponded to pharmaceuticals and were detected in volunteer C. Although the first-generation antihistaminic chlorphenamine (2a) was also observed in the rest of the volunteers, the concentration in volunteer C was at least 6 times higher than in the rest. It could be pinpointed that chlorphenamine is not only used as an antihistaminic but also found mixed with medicines to treat coughs and colds. As for the other annotated compounds, pidotinod is an immunostimulant that reinforces the respiratory system and epithienamycin and PS-6/penicillin F are antibiotics. It could be highlighted that the four pharmaceuticals were detected in the same volunteer and, as can be seen in Figure S6 in SI, the signal of those four compounds in the blanks is negligible compared to the signal in volunteer C samples. Additionally, the fragmentation spectra interpretation based either on the comparison with in silico fragmentation or mzCloud (see Figure S6 in SI), is in good accordance with the structure of the suspect. In the case of feature 4, neither mass spectra nor RTI criteria could differentiate between PS-6 and penicillin F isomers, limiting its annotation to level 3.

In the literature, few works are found for SNTS in urine samples. The work of Liu et al., for instance, was focused on analytical and data-processing approaches for PFAS rather than the application to real samples [70]. The work by Plassmann and coauthors [33] was focused on method development which was afterwards applied to a pooled urine sample (there was no mention of how many subsamples were used). MEtP, MBuP, MBnP, ethylosylamide, EtP, triglyme and BP3 were quantified through target analysis while 9 additional compounds were tentatively identified, including genistein, daidzein and cotinine in common with the present work [33]. As far as we know, the most complete work in terms of epidemiological application of SNTS in urine samples is a work developed by Caballero-Casero and coworkers. In that work, the presence of chemicals of emerging concern (CECs) in Flemish adolescents (25 female and 25 male) was screened, where 45 CECs were identified in levels 1–3 [71]. Compared to our work, they were able to identify compounds such as EtP or BPS that we could quantify by target analysis using LC-QqQ but not in the SNTS approach. On the contrary, other compounds such as the UV filter BP3 or the most common bisphenol, BPA, were not found in the mentioned work. One major difference between the two analytical protocols is that, in the present work, a hydrolysis step was performed so that we could not determine which compounds were present as phase II metabolites. This limitation will be tackled in future works by comparison of the results obtained with and without the hydrolysis step.

Finally, with the aim of improving our SNTS approach, a larger number of suspect lists could be introduced to increase the number of annotated compounds, as well as the removal of the compulsory mzCloud filter. However, the number of features annotated at levels 3, 4 and 5 will increase due to the impossibility of distinguishing among different candidates. Besides, the peak intensity value could be diminished but some filters that help on the selection of Lorentzian peaks would be compulsory in order to reduce noise. Lastly, a filtering column could be placed in the LC-qOrbitrap in order to delay the interferences coming from the system, so that we could diminish the ratio that takes into account the signal in the blanks. With that action, we could annotate features that not only are in the samples but also come from the system (phthalates or polymers, mostly), but their area in the samples is not 10 times higher than in the blanks.

## Conclusions

In this work, a target analysis method using SPE-LC-QqQ for quantifying 24 diverse EDCs in human urine has been fully optimized, validated and further extended to SNTS by means of LC-qOrbitrap in the full MS–ddMS acquisition mode. For the latter, a suspect list of 3826 candidates and a non-target analysis approach of compounds containing either Cl, Br and/or S atoms was used. As a result, 16 EDCs were quantified with the target analysis approach in the real urine samples, but the power of HRMS allowed the annotation of 62 suspects not included in the target analysis in this work. However, 12 quantified analytes were missed with the STNS approach, showing that although HRMS is a promising tool to screen xenobiotics in urine samples, the use of low-resolution mass analysers is still complementary due to the better limits of detection obtained. Moreover, the results obtained by the SPE-LC-qOrbitrap for the SNTS should be compared with those of other approaches, including the elimination of the hydrolysis step or even avoiding sample treatment in approaches such as “dilute-and-shoot.” The first action would allow the identification of phase II metabolites, while minimizing sample treatment would avoid potential losses during the analytical procedure. Nevertheless, other problems will arise, such as difficulties in annotation of glucuronide metabolites and signal suppression due to strong matrix effect in complex matrices, among others. For the SNTS, its extension to (semi)quantitative analysis should also be validated in future works. Moreover, problems related to signal drift and RT alignment in sequences where a large number of samples are processed should be assessed, as well as establishing more homogeneous criteria regarding QC/QA. Last but not least, taking into account the few works in the literature tackling SNTS in human biofluids compared to environmental samples, further studies are compulsory in this field of application in order to develop effective SNTS methods that would help in decoding the human exposome.

## Supplementary Information

Below is the link to the electronic supplementary material.

Supplementary file1 (PDF 1339 KB)

## Declarations

The study was approved by the Ethics Commission for Research and Teaching of the University of the Basque Country (CEISH-UPV/EHU, BOPV 32, 17/2/2014 M10 2021 124 and CEIAB-UPV/EHU, BOPV 32, 14/2/14, M30 2021 158), and all the experiments were performed in accordance with ethical standards.

Informed consent of all individual participants who provided urine samples was obtained prior to conducting the experiments.
