# Supplementary material for: From target analysis to suspect and non-target screening of endocrine-disrupting compounds in human urine
Source: Anal Bioanal Chem. 2022 Jul 29;414(23):6855–69. doi: 10.1007/s00216-022-04250-w (PMC9436830; doi:10.1007/s00216-022-04250-w)
Supplement: Supplementary file 1 — Supplementary file1 (PDF 1339 KB) [file 216_2022_4250_MOESM1_ESM.pdf]

From target analysis to suspect and non-target screening of endocrine disrupting compounds in human urine

Mikel Musatadi (corresponding author)

Department of Analytical Chemistry, University of the Basque Country (UPV/EHU), 48940 Leioa, Basque Country, Spain. Research Centre for Experimental Marine Biology and Biotechnology, University of the Basque Country (UPV/EHU), 48620 Plentzia, Basque Country, Spain. ORCID: 0000-0002-8328-5181

E-mail address: mikel.musatadi@ehu.eus

Claudia Caballero

Department of Analytical Chemistry, University of the Basque Country (UPV/EHU), 48940 Leioa, Basque Country, Spain.

Leire Mijangos

Department of Analytical Chemistry, University of the Basque Country (UPV/EHU), 48940 Leioa, Basque Country, Spain. Research Centre for Experimental Marine Biology and Biotechnology, University of the Basque Country (UPV/EHU), 48620 Plentzia, Basque Country, Spain. ORCID: 0000-0003-1872-4624

Ailette Prieto

Department of Analytical Chemistry, University of the Basque Country (UPV/EHU), 48940 Leioa, Basque Country, Spain. Research Centre for Experimental Marine Biology and Biotechnology, University of the Basque Country (UPV/EHU), 48620 Plentzia, Basque Country, Spain. ORCID: 0000-0002-4660-7188

Maitane Olivares

Department of Analytical Chemistry, University of the Basque Country (UPV/EHU), 48940 Leioa, Basque Country, Spain. Research Centre for Experimental Marine Biology and Biotechnology, University of the Basque Country (UPV/EHU), 48620 Plentzia, Basque Country, Spain. ORCID: 0000-0001-7047-3055

Olatz Zuloaga

Department of Analytical Chemistry, University of the Basque Country (UPV/EHU), 48940 Leioa, Basque Country, Spain. Research Centre for Experimental Marine Biology and Biotechnology, University of the Basque Country (UPV/EHU), 48620 Plentzia, Basque Country, Spain. ORCID: 0000-0002-3614-6293

CONTENTS

2. MATERIAL AND METHODS

2.1 Reagents and solutions

2.2 Development of the target method by UHPLC-ESI-QqQ

2.2.1 Sample treatment

2.2.2 UHPLC-ESI-QqQ analysis

2.2.3 Target method validation

2.3 Suspect and non-target screening by HRMS

2.3.1 UHPLC-HESI-qOrbitrap analysis

2.3.2 Data processing

2.4 Analysis of real urine samples

FIGURES

Figure S1 – Boxplots of the (a) recoveries and (b) matrix effects at detection for the elution solvents tested with Oasis HLB.

Figure S2 – SPE elution profile (3 – 12 mL) for the 24 EDCs using MeOH:Acetone 50:50 (v/v) as solvent.

Figure S3 – (a) Experimental (top) and mzCloud (down) mass spectrum for BP3 in the positive ionization mode and (b) experimental mass spectrum for BP3 in the negative ionization mode. Green dots indicate match either with the (a) reference spectrum or with the (b) *in-silico* prediction (b).

Figure S4 – (a) Experimental mass spectrum obtained for a standard of 50 ng g^{-1} concentration of triclosan and (b) mass spectra for triclosan obtained in mzCloud library

Figure S5 – PCA of the compounds (PC1 versus PC2) identified by suspect screening in the real samples at levels 1-3 in the positive mode: (a) scores plot and (b) loadings plot.

Figure S6 – Chromatograms and mass spectra tentatively annotated by non-target screening of Cl, Br o S containing compounds in volunteers' samples. Chromatogram for tentatively identified chlorpheniramine (a), comparison of experimental (top) and mzCloud (down) mass spectra for tentatively identified

chlorpheniramine (b), chromatogram for tentatively identified pidotimod (c), experimental mass spectra for tentatively identified pidotimod (d), chromatogram for tentatively identified epithienamycin (e), experimental mass spectra for tentatively identified epithienamycin (f), chromatogram for tentatively identified PS-6 or penicillin F (g) and experimental mass spectra for tentatively identified PS-6 (h) or mass spectra for tentatively identified penicillin F (i). Green dots in mass spectra indicate either match with mzCloud spectra (b) or *in-silico* match with the tentative structure (d, f, h, i).

TABLES

Table S1 - Information and physicochemical properties (obtained from Chemicalize) of the analytes and the surrogates included in the target analysis method, additional analytes introduced in suspect and non-target screening and RTI model analytes.

Table S2 - Mass spectrometry conditions for the target analysis of the 24 EDCs and 4 surrogates by LC-QqQ at pH 2.5 and 10.5, including the ionization mode, retention time (RT), *m/z* fragments, fragmentor voltage and collision energy (CE) for each compound.

Table S3 - Mass spectrometry conditions for the analysis by LC-qOrbitrap at pH 2.5 and 10.5 including the ionization mode, retention time (RT), precursor and product ions (*m/z*), as well as the instrumental limit of identification (iLOQ, ng g^{-1}) and the MS2 match for each compound.

Table S4 - Instrumental (iLOQ, ng g^{-1}) and procedural (pLOQ, ng g^{-1}) limits of quantification, upper limits (ng g^{-1}) and determination coefficients (r^2) of the calibration curves for LC-QqQ and LC-qOrbitrap at pH 2.5 and pH 10.5.

Table S5 - Average (n=5) absolute (%) and apparent (%) recoveries and relative standard deviations (RSDs, %) obtained at the (a) low (3 ng g^{-1}), (b) medium (6 ng g^{-1}) and (c) high (30 ng g^{-1}) spiking levels for SPE-LC-QqQ at pH 2.5 and 10.5. Surrogate used for apparent recovery calculation is included when applicable.

Table S6 – Compounds annotated at the volunteers´ samples at levels 1-5 using suspect and non-target screening by SPE-LC-qOrbitrap.

2. MATERIAL AND METHODS

2.1 Reagents and solutions

Dimethyl sulfoxide (DMSO), potassium chloride (KCl, 99.0 %) and orthophosphoric acid (H_3PO_4, 85 %) were purchased from Applichem Panreac (Barcelona, Spain); Milli-Q water (H_2O) and methanol (MeOH, UHPLC-MS quality) from Scharlab (Barcelona, Spain); sodium chloride (NaCl, 99.5 %), citric acid (99.5 – 100.5 %), sodium bicarbonate ($NaHCO_3$, ≥ 99.5 %) and hydrochloric acid (HCl, 36 %) from Merck (Darmstandt, Germany); urea (> 98.0 %), ascorbic acid (> 99 %), potassium phosphate (K_3PO_4, > 98 %), creatinine (anhydrous, ≥ 98 %), β-glucuronidase enzyme (β-glucuronidase from Helix Pomatia, $2 \cdot 10^6$ units g^{-1}), ammonium acetate (NH_4OAc, ≥ 99.99 %) from Sigma-Aldrich (Darmstadt, Germany); sodium hydroxide (NaOH), ammonia (NH_4OH in solution, 25 %) and formic acid (HCOOH, ≥ 98.0 %) from Honeywell Fluka (Muskegon, MI, USA); Milli-Q quality H_2O (< 0.05 µS cm^{-1}) using Millipore 185 from Millipore (Burlington, MA, USA); anhydrous MeOH, acetonitrile (AcN), ethyl acetate (EtOAc), dichloromethane (DCM) and acetone from ChromAR HPLC (Macron Fine Chemicals, Avantor, Radnor Township, PA, USA) and were employed as reagents and/or solvents. Nitrogen gas (N_2, 99.999 %) was provided by Air Liquide (Madrid, Spain).

For the preparation of synthetic urine, 3.80 g KCl, 8.50 g NaCl, 24.50 g urea, 1.03 g citric acid, 0.34 g ascorbic acid, 1.18 g potassium phosphate, 1.40 g creatinine, 0.64 g NaOH, 0.45 g $NaHCO_3$ and 0.28 mL H_2SO_4 were dissolved in 500 mL Milli-Q H_2O and stirred for 60 minutes [1]. Regarding the rest of the solutions, β-glucuronidase (20000 units mL^{-1}) was obtained by dissolving the enzyme in 1 M NH_4OAc (pH 5.0) solution, which was prepared with NH_4OAc and HCl. Phosphate buffer ($H_3PO_4/H_2PO_4^-$, 0.1 M, pH 2.0) was prepared using H_3PO_4 and NaOH, and formic acid/formate buffer ($HCOOH/HCOO^-$, 1 M, pH 2.0) employing HCOOH and NaOH. Finally, 0.1 % HCOOH (pH 2.5) and 0.05 % NH_4OH (pH 10.5) in UHPLC H_2O and MeOH were prepared as well.

2.2 Development of the target method by UHPLC-ESI-QqQ

2.2.1 Sample treatment

For SPE recovery calculation, synthetic urine samples were spiked (A) before (n=3) and (B) after (n=3) the procedure to get 100 ng g^{-1} in the final extract, and it was expressed following equation 1 (Eq. 1).

$$\text{Recovery (\%)} = \frac{\text{Analytical signal in A}}{\text{Analytical signal in B}} \cdot 100 \text{ (Eq. 1)}$$

For estimating the matrix effect, samples spiked (B) after SPE were compared to a (C) reference point of 100 ng g^{-1} injected in triplicate, due to the estimated preconcentration factor of 5. It was then expressed as equation 2 (Eq. 2) [2]. A matrix effect value above 0 indicates signal enhancement, while a value below 0 shows signal suppression.

$$\text{Matrix effect (\%)} = \left(\frac{\text{Analytical signal in B}}{\text{Analytical signal in C}} - 1\right) \cdot 100 \text{ (Eq. 2)}$$

2.2.2 UHPLC-ESI-QqQ analysis

As for the gradient elution, the starting composition was held at 20 % B for 1 minute and changed to 45 % in another minute. After maintaining that composition for 1 minute, the organic modifier was incremented to 50 % in 1.5 minutes, to 55 % in other 1.5 minutes and to 80 % in 2 minutes. Finally, it was changed to 90 % in 3 minutes, maintaining it for 5 minutes. In order to reset the starting conditions, the B line composition was reduced from 90 % to 20 % in 3 minutes and kept in that starting composition for 6 minutes ensuring proper equilibration, being the total run time 25 minutes.

The detection and quantification of the 24 target compounds and 4 surrogates were carried out using the Dynamic MRM (DMRM) acquisition mode, both at positive and negative ionization. The capillary voltage used was 3000 V, while a flow of 12 L min^{-1} of N_2 was required for the evaporation of the mobile phase. The temperature and nebulizing pressure were respectively 350 °C and 30 psi. Those parameters were selected following the experience of the research group and were not further optimized. The fragmentor´s electric voltage (20-200 V), *m/z* transitions and the collision energy (5-60 eV) for each transition were optimized using the MassHunter Optimizer software by injection of individual compounds without column. Optimum conditions, as well as the precursor and product ions used, are summarized in Table S2 for each target compound. Finally, instrumental operation, data acquisition, and peak integration were performed with the MassHunter Workstation Software (Acquisition, Quantitative and Qualitative Analysis for QqQ, Version 10.0, Agilent Technologies).

2.2.3 Target method validation

An external calibration of the target compounds was prepared in the 0.5-200 ng g^{-1} range in H_2O:DMSO (80:20, v/v). Low calibration points (0.5 - 50 ng g^{-1}) were injected in triplicate to calculate iLOQs, which was defined

for each analyte as the lowest concentration level that fitted the calibration curve with a relative standard deviation (RSD %) and systematic error lower than 30 %. Using the calibration curves, linearity ranges were defined considering the determination coefficients (r^2) of the curves built between iLOQ and upper limit. The latter was established as the highest concentration possible that provided a r^2 value closest to 1 avoiding quadratic fitting of the calibration curves.

Absolute recoveries (%) were determined as the average (n=5) ratio of the concentration obtained from the calibration curve and real the concentration used for spiking synthetic urine at the three spiking levels in the three consecutive days. In the case of apparent recoveries (%), they were estimated after correction of the absolute recoveries with the corresponding surrogate. [$^2H_5$]-BP3, [$^2H_{16}$]-BPA, [$^2H_4$]-BnBuP and [$^{13}C_4$]-DEHP were studied as possible surrogates. Moreover, pLOQs were estimated considering the corresponding iLOQ, sample pretreatment (preconcentration factor of 5) and absolute recovery at the closest spiked concentration-level.

Finally, the intra-day precision of the method was estimated by calculating the RSD for the replicates (n=5) of spiked samples, while inter-day precision was determined by the replicates of spiked samples (n=5) in three consecutive days for each spiking level. As for the criteria, RSD values below 35 % were considered as satisfactory considering the nature of the analytes, the complexity of the matrix and the exhaustive analytical procedure. Both intra- and inter-day precision were statistically evaluated using ANOVA at a 95 % confidence-level as well.

2.3 Suspect and non-target screening by HRMS

2.3.1 UHPLC-HESI-qOrbitrap analysis

The qOrbitrap mass analyzer was externally calibrated every three days using Pierce LTQ ESI (Thermo Fisher Scientific) calibration solutions. Measurements were performed at negative and positive ionization modes in the Full scan - data dependent MS2 (Full MS-ddMS2) discovery acquisition mode in the *m/z* 70 – 1050 Da range. In that acquisition mode, after a complete scan at 70,000 full width at half maximum (FWHM) resolution at *m/z* 200, three scans were performed in the *m/z* 100 – 600 Da range at 17,500 FWHM at *m/z* 200 with an isolation window of 3.0 *m/z* with a stepped collision energy (SCE) of 10, 45 and 90 eV. The ddMS2 scans were performed with an automatic intensity threshold and dynamic exclusion. Automatic Gain Control (ACG) target was set at $5{\cdot}10^4$ and its minimum was set at $8{\cdot}10^3$. As for the HESI parameters, the spray voltages

were set at 3.2 kV for positive and 3.5 kV for negative ionization modes. For positive ionization, the capillary temperature was maintained at 320 °C, the sheath gas at 40 arbitrary units (au), the auxiliary gas at 15 au and 310 °C, and the sweep gas at 1 au. For negative ionization, the capillary temperature was set at 300 °C, the sheath gas at 40 au, the auxiliary gas at 15 au and 280 °C, and the sweep gas at 1 au. Xcalibur 4.4 (Thermo Fischer Scientific) was used for external instrumental control.

2.3.2 Data processing

After peak picking, the Compound Discoverer 3.2 provided the features that, according to their MS1 (an exact mass with an error lower than 5 ppm and isotopic profile with at least a 70 % fitting), matched with one or several candidates in the suspect lists. In order to reduce the number of features, only those features which had at least one candidate in mzCloud library (Thermo-Fischer Scientific) were considered. When not all the suspects for a feature had a fragmentation pattern (MS2) in mzCloud, *in-silico* fragmentation was performed for those candidates using the MassFrontiers program (Thermo-Fischer Scientific) implemented in Compound Discoverer 3.2 to avoid false positives.

For mzCloud library match, a fitting higher than 70 % was settled. For *in-silico* fragmentation instead, at least a 70 % of the major fragments should be explained when the fragmentation mass spectra contained more than two fragments, otherwise the major fragment should be explained. Finally, RT was used for confirmation. When the pure standard was available, a deviation of ± 0.1 min was considered and, if not, the experimental RT should be within the error window calculated using the RTI platform (http://rti.chem.uoa.gr/). Calibrants included in Table S1 were used to build the RT model [3]. The annotation confidence levels are also indicated in Figure 1 and were set as follows [4]: level 1 for suspects whose RT, MS1 and MS2 could be satisfactorily explained using a pure standard; level 2a was assigned when fragmentation spectra could be explained against a match with a fragmentation spectrum in mzCloud library and the experimental RT passed the RTI model criteria; level 2b when fragmentation spectra was satisfactorily explained using *in-silico* fragmentation and the experimental RT passed the RTI criteria; level 3 when different positional isomers passed the criteria stablished for either level 2a or level 2b; level 4 when only the chemical formula could be stablished and, finally, level 5 when not even the molecular formula could be assigned.

For avoiding annotation of endogenous metabolites, the list obtained from HMDB was used. Instead of using it as exclusion list, it was employed as inclusion list to avoid discarding features that contained more than one

candidate, from which one or more could be endogenous but also had exogenous candidates. If a feature had only one candidate, and it was in the HMDB list, it was discarded. For the rest of the cases (features having endogenous and exogenous candidates), the workflow was applied, and if an endogenous candidate passed the stablished criteria, the feature was then discarded.

2.4 Analysis of real urine samples

First urine void for each volunteer (30 mL approx.) was collected in a glass vial, stored at 4 °C and analysed within 24 hours after collection. Briefly, urine samples were tempered to room temperature and 1 mL aliquots (n=5) were spiked with isotopically labelled standards at 20 ng g^{-1} prior optimized sample treatment. Final extracts were randomly injected at both LC-QqQ for target analysis and LC-qOrbitap to perform SNTS, alongside with procedural blanks spiked with the isotopically labelled standards, clean MeOH samples every 5 injections and a reference point of 50 ng g^{-1} every 10 injections. Analyte quantification by target analysis and suspect identification by SNTS were carried out following the previously mentioned conditions and criteria.

FIGURES

(a)

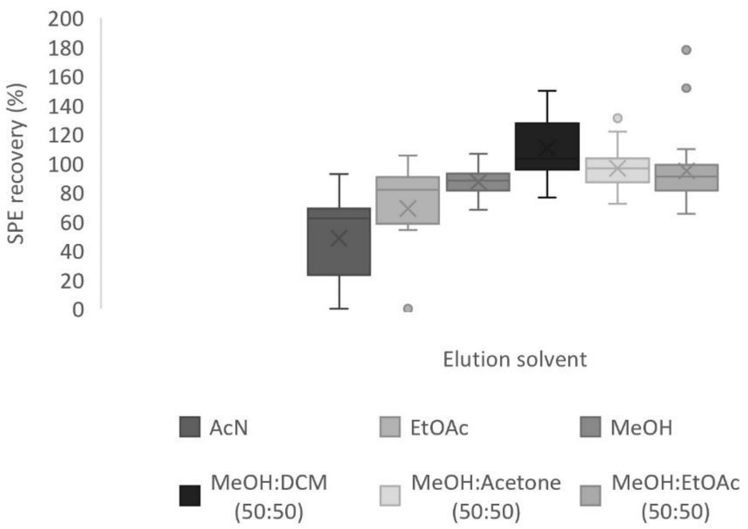

(b)

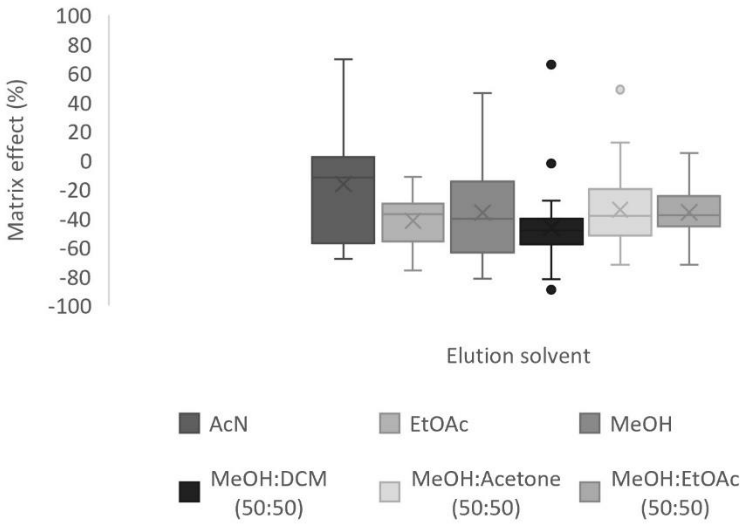

Figure S1 – Boxplots of the (a) recoveries and (b) matrix effects at detection for the elution solvents tested in the RP-SPE approach using Oasis HLB.

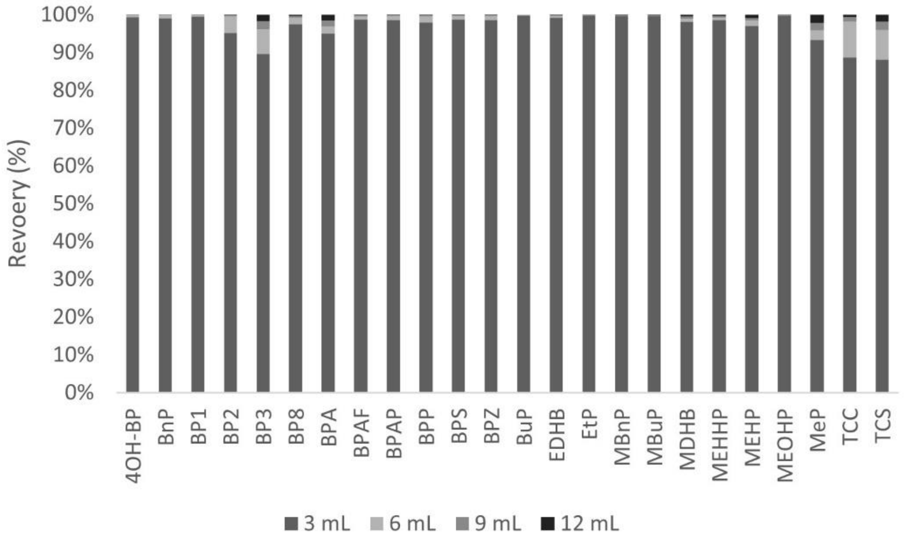

Figure S2 – SPE elution profile with Oasis HLB for the 24 EDCs using MeOH:Acetone 50:50 (v/v) as elution solvent.

(a)

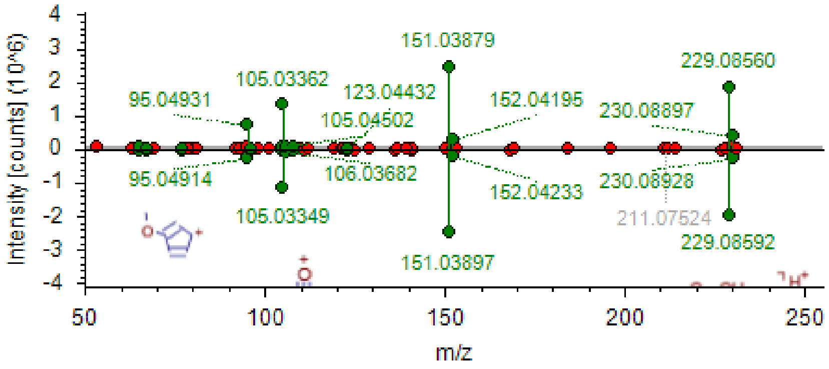

(b)

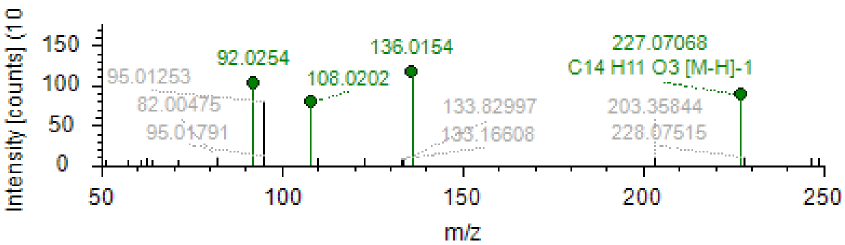

Figure S3 – (a) Experimental (top) and mzCloud (down) mass spectrum for BP3 in the positive ionization mode and (b) experimental mass spectrum for BP3 in the negative ionization mode. Green dots indicate match either with the (a) reference spectrum or with the (b) *in-silico* prediction.

(a)

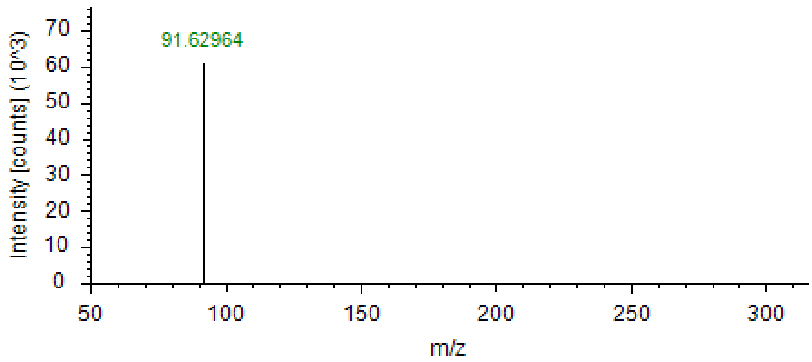

(b)

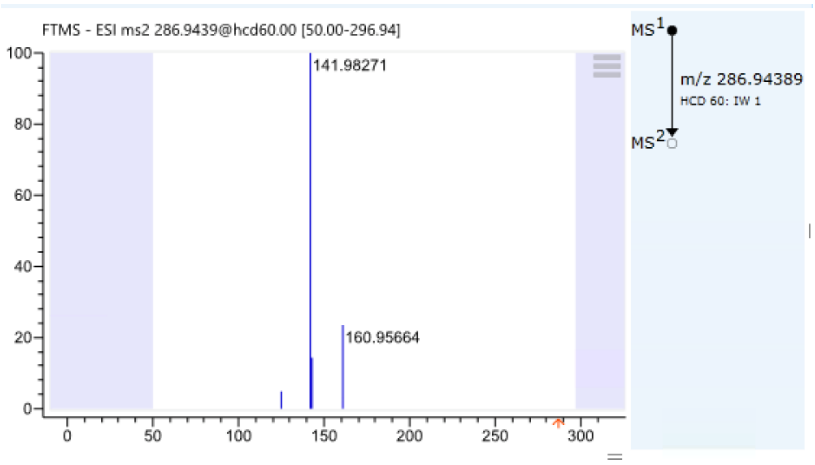

Figure S4 – (a) Experimental mass spectrum obtained for a standard of 50 ng g^{-1} concentration of triclosan and (b) mass spectra for triclosan obtained in mzCloud library.

(a)

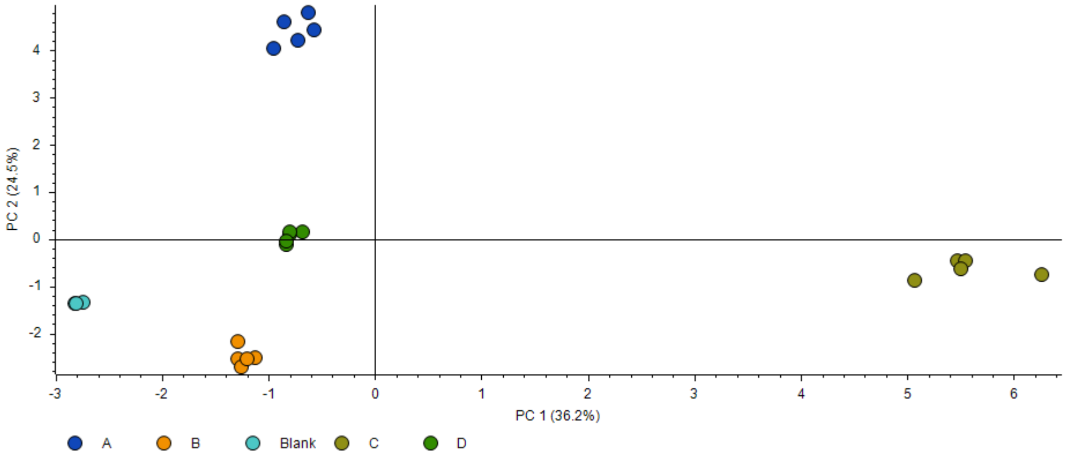

(b)

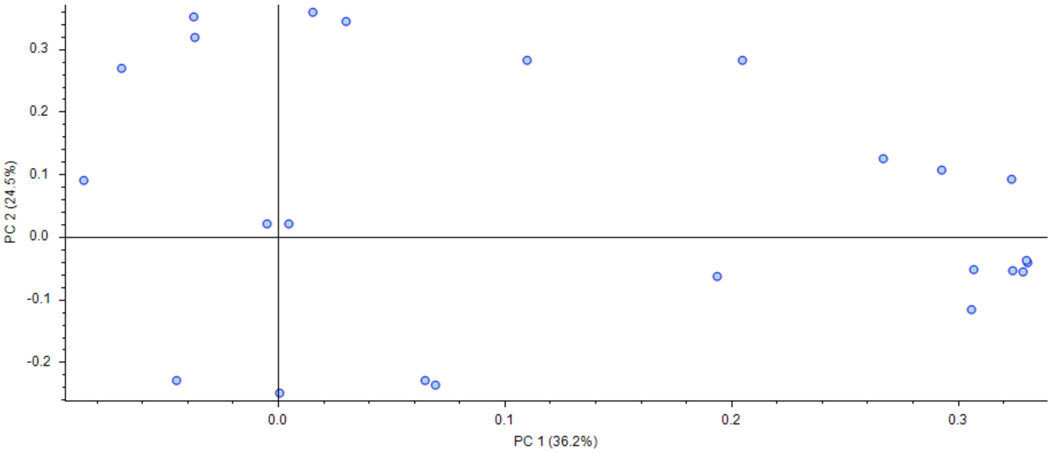

Figure S5 – (a) Scores plot and (b) loadings of the compounds (PC1 versus PC2) identified by suspect screening in the real samples at levels 1-3 in the positive mode.

(a)

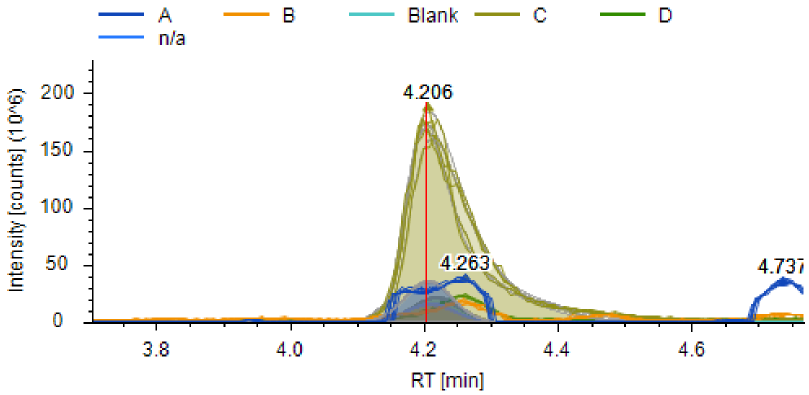

(b)

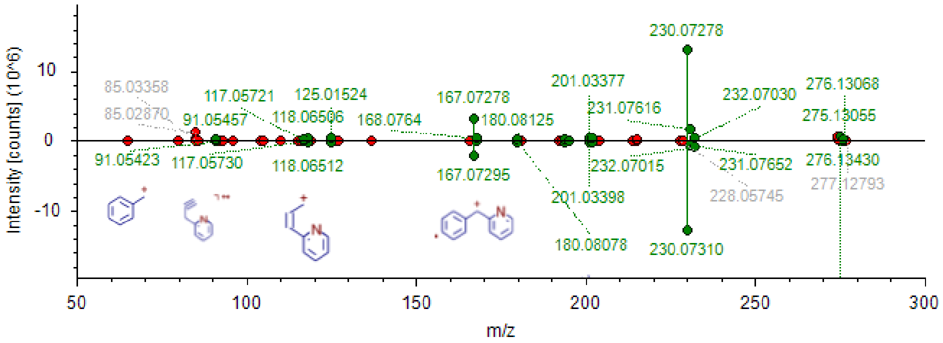

(c)

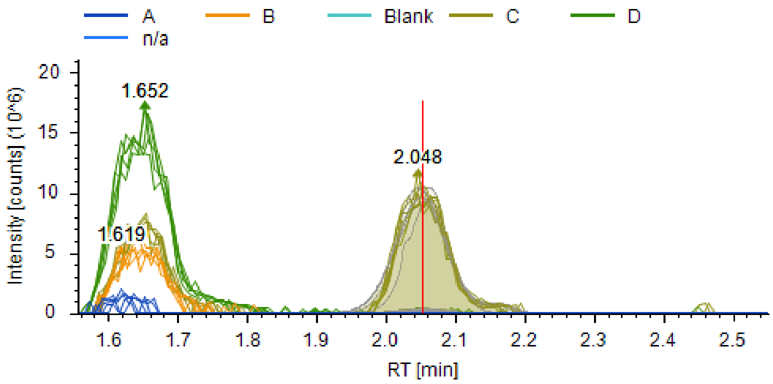
A
n/a
B
Blank
C
D
Intensity [counts] (10^6)
1.652
2.048
1.619
RT [min]

(d)

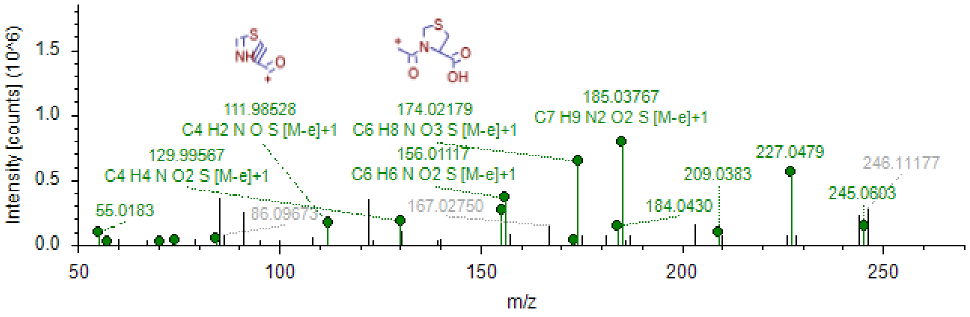
Intensity [counts] (10^6)
111.98528
C4 H2 N O S [M-e]+1
174.02179
C6 H8 N O3 S [M-e]+1
185.03767
C7 H9 N2 O2 S [M-e]+1
129.99567
C4 H4 N O2 S [M-e]+1
156.01117
C6 H6 N O2 S [M-e]+1
227.0479
246.11177
209.0383
245.0603
55.0183
86.09673
167.02750
184.0430
m/z

(e)

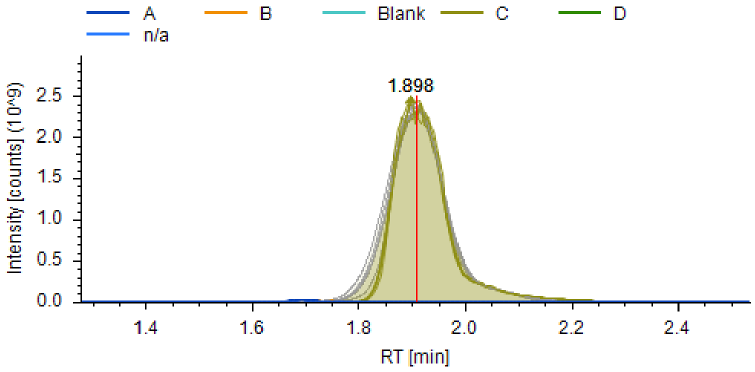
A
n/a
B
Blank
C
D
Intensity [counts] (10^9)
1.898
RT [min]

(f)

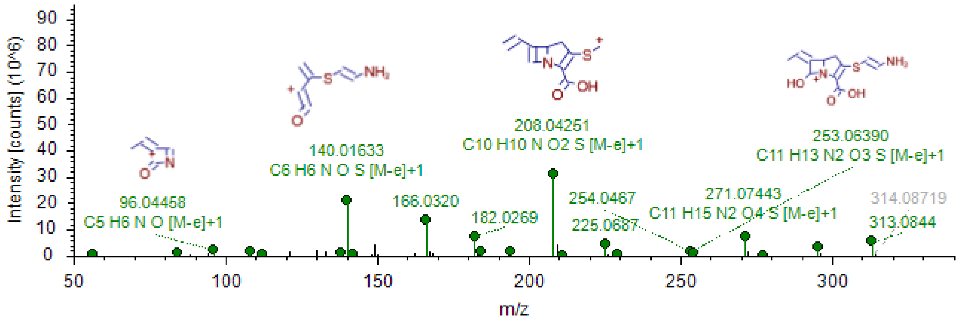
Intensity [counts] (10^6)
208.04251
C10 H10 N O2 S [M-e]+1
253.06390
C11 H13 N2 O3 S [M-e]+1
140.01633
C6 H6 N O S [M-e]+1
96.04458
C5 H6 N O [M-e]+1
166.0320
182.0269
254.0467
225.0687
271.07443
C11 H15 N2 O4 S [M-e]+1
314.08719
313.0844
m/z

(g)

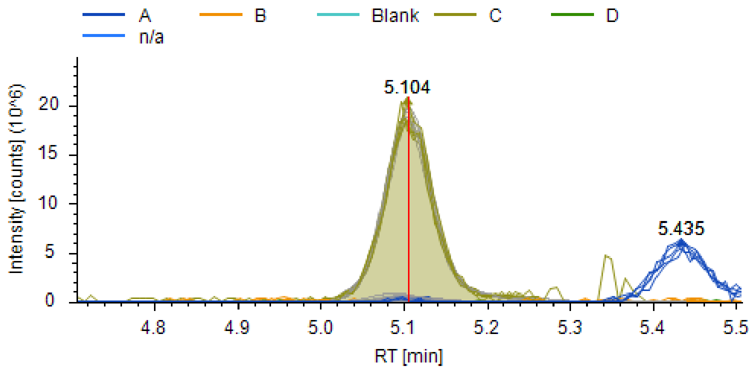

(h)

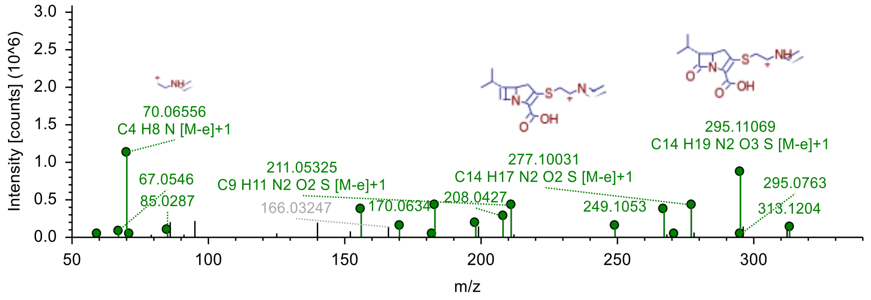

(i)

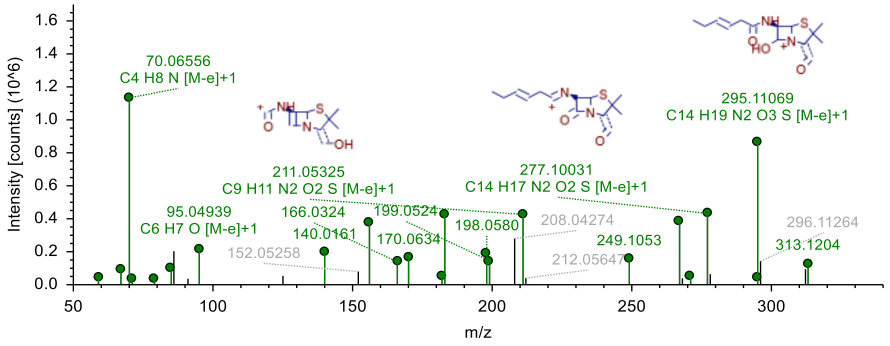

Figure S6 – Chromatograms and mass spectra tentatively annotated after non-target screening of Cl, Br o S containing compounds in volunteers' samples. Chromatogram for (a) tentatively identified chlorpheniramine, (b) comparison of experimental (top) and mzCloud (down) mass spectra for tentatively identified chlorpheniramine, (c) chromatogram for tentatively identified pidotimod, (d) experimental mass spectra for tentatively identified pidotimod, (e) chromatogram for tentatively identified epithienamycin, (f) experimental mass spectra for tentatively identified epithienamycin, (g) chromatogram for tentatively identified PS-6 or penicillin F and (h) experimental mass spectra for tentatively identified PS-6 or (i) mass spectra for tentatively identified penicillin F. Green dots in mass spectra indicate either (b) match with mzCloud spectra or (d, f, h, i) in-silico match with the tentative structure.

TABLES

Table S1 - Information and physicochemical properties (obtained from Chemicalize) of the analytes and the surrogates included in the target analysis method, additional analytes introduced in suspect and non-target screening and RTI model analytes.

COMPOUND	Abbreviation	CAS number	Commercial vendor	Purity (%)	Solvent	Molecular formula	Exact mass (Da)	pKa	Log D (2.5; 10.5)
				Benzophenones					
4,4'-bis(dimethylamino)benzophenone	4,4-DMA-BP	90-94-8	Sigma-Aldrich	99	MeOH	$C_{17}H_{20}N_2O$	268.15756	3.2; 3.7	1.7; 3.6
4-Hydroxybenzophenone [a]	4-OH-BP	1137-42-4	Sigma-Aldrich	100	MeOH	$C_{13}H_{10}O_2$	198.06808	7.8	3.1; 1.2
4-Methylbenzophenone	4-MeBP	134-84-9	Sigma-Aldrich	99	MeOH	$C_{14}H_{12}O$	196.08882	[b]	3.9; 3.9
4-Phenylbenzophenone	4-PhBP	2128-93-0	Sigma-Aldrich	99	MeOH	$C_{19}H_{14}O$	258.10447	[b]	5.1; 5.1
Benzophenone-1 [a]	BP1	131-56-6	Sigma-Aldrich	99	MeOH	$C_{13}H_{10}O_3$	214.06299	7.1; 8.7	3.5; 0.2
Benzophenone-2 [a]	BP2	131-55-5	Sigma-Aldrich	99	MeOH	$C_{13}H_{10}O_5$	246.05282	6.8; 7.4; 8.4; 9.0	3.5; -3.1
Benzophenone-3 [a]	BP3	131-57-7	Sigma-Aldrich	100	MeOH	$C_{14}H_{12}O_3$	228.07864	7.1	3.6; 1.6
Benzophenone-8 [a]	BP8	131-53-3	Sigma-Aldrich	98	MeOH	$C_{13}H_{10}O$	244.07356	6.8; 7.4	4.0; -0.1
				Bisphenols					
Bisphenol A [a]	BPA	80-05-7	Sigma-Aldrich	98	MeOH	$C_{15}H_{16}O_2$	228.11503	9.8; 10.4	4.1; 3.5
Bisphenol AF [a]	BPAF	1478-61-1	Sigma-Aldrich	98	MeOH	$C_{15}H_{10}F_6O_2$	336.05850	9.7; 9.1	4.8; 3.5
Bisphenol AP [a]	BPAP	1571-75-1	Sigma-Aldrich	99	MeOH	$C_{20}H_{18}O_2$	290.13068	9.7; 10.3	5.2; 4.5
Bisphenol P [a]	BPP	2167-51-3	Sigma-Aldrich	97	MeOH	$C_{24}H_{16}O_2$	346.19328	9.8; 10.4	6.7; 6.2
Bisphenol S [a]	BPS	80-09-1	Sigma-Aldrich	98	MeOH	$C_{12}H_{10}O_4S$	250.02998	7.4; 8.0	2.3; -1.5
Bisphenol Z [a]	BPZ	843-55-0	Sigma-Aldrich	98	MeOH	$C_{18}H_{20}O_2$	268.14633	9.8; 10.4	4.9; 4.4
Bisphenol A glucuronide [c]	BPA-G	267244-08-6	Sigma-Aldrich	99	MeOH	$C_{21}H_{24}O_8$	404.14712	3.3; 10.1; 12.2; 13.3; 14.8	1.9; -1.7
				Parabens					
Benzyl paraben [a]	BnP	94-18-8	Sigma-Aldrich	97	MeOH	$C_{14}H_{12}O_3$	228.07864	8.5	3.4; 2.0
Butyl paraben [a]	BuP	94-26-8	Sigma-Aldrich	99	MeOH	$C_{11}H_{14}O_3$	194.09429	8.5	3.0; 1.6
Ethyl 3,4-dihydroxybenzoate [a]	EDHB	3943-89-3	Sigma-Aldrich	98	MeOH	$C_9H_{10}O_4$	182.05790	8.6; 12.2	1.7; 0.4
Ethyl paraben [a]	EtP	120-47-8	Sigma-Aldrich	99	MeOH	$C_9H_{10}O_4$	166.06299	8.5	2.0; 0.3
Methyl 3,5-dihydroxybenzoate [a]	MDHB	2150-44-9	Sigma-Aldrich	97	MeOH	$C_8H_8O_4$	168.04226	8.9; 10.3	1.4; 0.1
Methyl paraben [a]	MeP	99-76-3	Sigma-Aldrich	98	MeOH	$C_8H_8O_3$	152.04734	8.5	1.7; -0.2

Table S1 – Continuation.

COMPOUND	Abbreviation	CAS number	Commercial vendor	Purity (%)	Solvent	Molecular formula	Exact mass (Da)	pKa	Log D (2.5; 10.5)
				Phthalates					
Mono-(2-ethyl-5-hydroxyhexyl) phthalate [a]	MEHHP	40321-99-1	Sigma-Aldrich	100	MeOH	$C_{16}H_{22}O_5$	294.14672	-1.7; 3.1	2.9; -0.3
Mono-[(2RS)-2-Ethyl-5-oxohexyl phthalate [a]	MEOHP	40321-98-0	Sigma-Aldrich	100	MeOH	$C_{16}H_{20}O_5$	292.13107	3.1	3.0; -0.3
Mono-2-Ethylhexyl phthalate [a]	MEHP	4376-20-9	Sigma-Aldrich	98	MeOH	$C_{16}H_{22}O_4$	278.15181	3.1	4.4; 1.1
Mono-benzyl phthalate [a]	MBnP	2528-16-7	Sigma-Aldrich	97	MeOH	$C_{15}H_{12}O_4$	256.07356	3.1	3.1; -0.2
Mono-butyl phthalate [a]	MBuP	131-70-4	Sigma-Aldrich	95	MeOH	$C_{12}H_{14}O_4$	222.08921	3.1	2.7; -0.6
				Antibacterials and biocides					
Triclocarban [a]	TCC	101-20-2	Sigma-Aldrich	99	MeOH	$C_{13}H_9Cl_3N_2O$	313.97805	11.4	4.9; 4.9
Triclosan [a]	TCS	3380-34-5	Sigma-Aldrich	99	MeOH	$C_{12}H_7Cl_3O_2$	287.95116	7.7	5.0; 2.9
Acetamiprid		160430-64-8	Sigma-Aldrich	100	MeOH	$C_{10}H_{11}ClN_4$	222.06722	-0.3; 4.2	-0.5; 1.1
Alachlor		15972-60-8	Sigma-Aldrich	99.8	MeOH	$C_{14}H_{20}ClNO_2$	269.11826	[b]	3.6; 3.6
Ametryn		834-12-8	Sigma-Aldrich	98.6	MeOH	$C_9H_{17}N_5S$	227.12047	0.5; 6.7; 14.6	0.6; 2.6
Atrazine		1912-24-9	Fluka	99	MeOH	$C_8H_{14}ClN_5$	215.09377	4.2; 14.5; 15.8	0.7; 2.2
Boscalid		188425-85-6	Sigma-Aldrich	99.9	MeOH	$C_{18}H_{12}Cl_2N_2O$	342.03267	-0.1; 14.3	4.9; 4.9
Chloridazon		1698-60-8	Sigma-Aldrich	99.8	MeOH	$C_{10}H_8ClN_3O$	221.03559	-1.8	1.1; 1.1
Chloroxuron		1982-47-4	Sigma-Aldrich	98.8	MeOH	$C_{15}H_{15}ClN_2O_2$	290.08221	13.9	3.4; 3.4
Chlortoluron		15545-48-9	Sigma-Aldrich	99.5	MeOH	$C_{10}H_{13}ClN_2O$	212.07164	13.5	2.4; 2.4
Clomazone		81777-89-1	Sigma-Aldrich	98.3	MeOH	$C_{12}H_{14}ClNO_2$	239.07131	[b]	2.9; 2.8
Dichlorvos		62-73-7	Sigma-Aldrich	99	MeOH	$C_4H_7Cl_2O_4P$	219.94590	[b]	1.4; 1.4
Diethyl Toluamide		134-62-3	Sigma-Aldrich	98.8	MeOH	$C_{12}H_{17}NO$	191.13101	-0.9	2.5; 2.5
Dimethachlor		50563-36-5	Sigma-Aldrich	99.8	AcN	$C_{13}H_{18}ClNO_2$	255.10261	[b]	2.6; 2.6
Dimethoate		60-51-5	Sigma-Aldrich	99.8	MeOH	$C_5H_{12}NO_3PS_2$	228.99962	15.9	0.3; 0.3
Diuron		330-54-1	Fluka	98	MeOH	$C_9H_{10}Cl_2N_2O$	232.01702	13.2	2.5; 2.5
Ethion		563-12-2	Sigma-Aldrich	97.6	MeOH	$C_9H_{22}O_4P_2S_4$	383.98762	[b]	3.9; 3.9
Fenthion		55-38-9	Sigma-Aldrich	98.6	AcN	$C_{10}H_{15}O_3PS_2$	278.02002	[b]	3.8; 3.8
Flusilazole		85509-19-9	Sigma-Aldrich	100	Acetone	$C_{16}H_{15}F_2N_3Si$	315.10033	2.3	4.5; 4.7
Imidacloprid		138261-41-3	Sigma-Aldrich	99	MeOH	$C_9H_{10}ClN_5O_2$	255.05230	1.1; -0.1; 15.7	0.5; 0.5

Table S1 – Continuation.

COMPOUND	Abbreviation	CAS number	Commercial vendor	Purity (%)	Solvent	Molecular formula	Exact mass (Da)	pKa	Log D (2.5; 10.5)
Linuron		330-55-2	Sigma-Aldrich	99.7	AcN	$C_9H_{10}Cl_2N_2O_2$	248.01193	11.9	2.7; 2.7
Mecoprop		93-65-2	Sigma-Aldrich	99.6	MeOH	C10H11ClO3	214.03967	3.5	2.9; -0.5
Metazachlor		67129-08-2	Sigma-Aldrich	99.4	MeOH	$C_{14}H_{16}ClN_3O$	277.09819	2.3	2.7; 3.0
Metconazole		125116-23-6	Sigma-Aldrich	98.9	AcN	$C_{17}H_{22}ClN_3O$	319.14514	2.0; 13.7	3.5; 3.6
Methiocarb		2032-65-7	Sigma-Aldrich	99.8	MeOH	$C_{11}H_{15}NO_2S$	225.08235	14.8	3.1; 3.1
Metolachlor		51218-45-2	Sigma-Aldrich	97.6	MeOH	$C_{15}H_{22}ClNO_2$	283.13391	[b]	3.5; 3.5
Metribuzin		21087-64-9	Sigma-Aldrich	99	Acetone	$C_8H_4N_4OS$	214.08883	2.5	1.7; 2.0
Myclobutanil		88671-89-0	Sigma-Aldrich	99.6	MeOH	$C_{15}H_{17}ClN_4$	288.11417	2.3	3.5; 3.7
Oryzalin		19044-88-3	Sigma-Aldrich	99.1	MeOH	$C_{12}H_{18}N_4O_6S$	346.09471	9.6	2.3; 1.6
Pirimiphos-methyl		29232-93-7	Sigma-Aldrich	98.5	MeOH	$C_{11}H_{20}N_3O_3PS$	305.09630	-1.5; 5.1	1.1; 3.0
Prochloraz		67747-09-5	Sigma-Aldrich	98.6	MeOH	$C_{15}H_{16}Cl_3N_3O_2$	375.03081	2.6	3.4; 3.6
Propachlor		1918-16-7	Sigma-Aldrich	99.8	MeOH	$C_{11}H_{14}ClNO$	211.07639	[b]	2.4; 2.4
Propanil		709-98-8	Sigma-Aldrich	99.7	MeOH	$C_9H_9Cl_2NO$	217.00612	13.9	3.1; 3.1
Propiconazole		60207-90-1	Sigma-Aldrich	99.1	MeOH	$C_{15}H_{17}Cl_2N_3O_2$	341.06978	1.9	4.2; 4.3
Propyzamide		23950-58-5	Sigma-Aldrich	99.8	MeOH	$C_{12}H_{11}Cl_2NO$	255.02177	-1.5; 14.2	3.2; 3.2
Pyrazophos		13457-18-6	Sigma-Aldrich	99.8	MeOH	$C_{14}H_{20}N_3O_5PS$	373.08613	-0.6	3.1; 3.1
Quinmerac		90717-03-6	Sigma-Aldrich	99.9	MeOH	$C_{11}H_8ClNO_2$	221.02436	2.4; 3.6	2.6; -0.6
Simazine		122-34-9	Fluka	99	MeOH	$C_7H_{12}ClN_5$	201.07812	4.2; 14.8	0.2; 1.8
Tebuconazole		107534-96-3	Sigma-Aldrich	99.3	MeOH	$C_{16}H_{22}ClN_3O$	307.14514	2.0; 13.9	3.6; 3.7
Terbuthylazine		5915-41-3	Sigma-Aldrich	99.4	EtOH	$C_9H_{16}ClN_5$	229.10942	4.2; 14.2; 15.8	1.0; 2.5
Terbutryn		866-50-0	Sigma-Aldrich	99.1	MeOH	$C_{10}H_{19}N_5S$	241.13612	0.4; 6.7; 14.3; 15.9	0.9; 2.9
Thiacloprid		111988-49-9	Sigma-Aldrich	99.9	AcN	$C_{10}H_9ClN_4S$	252.02365	-0.2; 1.6	2.0; 2.1
Triadimenol		55219-65-3	Sigma-Aldrich	98.2	AcN	$C_{14}H_{18}ClN_3O_2$	295.10875	2.0; 13.2	3.2; 3.3
				Industrial chemicals					
2,4-Diethyl-9H-thioxanthen-9-one	DETX	82799-44-8	Sigma-Aldrich	99	MeOH	$C_{17}H_{16}OS$	268.09219	[b]	5.6; 5.6
2,4-Dinitrophenol		51-28-5	Sigma-Aldrich	99	MeOH	$C_6H_4N_2O_5$	184.01202	4.5	1.5; -0.4
2-Hydroxybenzothiazole	2-OH-BTA	934-34-9	Sigma-Aldrich	99.9	MeOH	C_7H_5NOS	151.00918	-1.3; 11.3	2.0; 1.9
2-isopropylthioxanthone	ITX	5495-84-1	Sigma-Aldrich	99	MeOH	$C_{16}H_{14}OS$	254.07654	[b]	4.9; 4.9

Table S1 – Continuation.

COMPOUND	Abbreviation	CAS number	Commercial vendor	Purity (%)	Solvent	Molecular formula	Exact mass (Da)	pKa	Log D (2.5; 10.5)
Benzothiazole		95-16-9	Sigma-Aldrich	97	MeOH	C_7H_5NS	135.01427	2.3	2.0; 2.1
Caffeine		58-08-2	Sigma-Aldrich	98	MeOH	$C_8H_{10}N_4O_2$	194.08038	-1.2	-0.5; -0.5
Cotinine		486-56-6	Sigma-Aldrich	98.5	MeOH	$C_{10}H_{12}N_2O$	176.09496	-1.8; 4.8	-0.8; 0.2
				PFAS					
Perfluorobutanesulfonic acid	PFBS	375-73-5	Sigma-Aldrich	98	MeOH	$C_4HF_9O_3S$	299.95027	-3.3	0.3; 0.3
Perfluorooctanesulfonamide	PFOSA	754-91-6	Dr. Ehrestofer	99.5	MeOH	$C_8H_2F_{17}NO_2S$	498.95348	3.4	4.8; 3.9
Perfluorooctanesulfonic acid	PFOS	1763-23-1	Sigma-Aldrich	97	MeOH	$C_8HF_{17}O_3S$	499.93749	-3.3	3.1; 3.1
				Pharmaceuticals					
Acetaminophen		103-90-2	Fluka	99.9	MeOH	$C_8H_9NO_2$	151.06333	9.5	0.9; -0.1
Diazepam		439-14-5	Sigma-Aldrich	>95	MeOH	$C_{16}H_{13}ClNCO$	284.07164	2.9	2.5; 3.1
Diclofenac		15307-86-5	Sigma-Aldrich	99	MeOH	$C_{14}H_{11}Cl_2NO_2$	295.01668	4	4.0; 0.7
Diphenhydramine		58-73-1	Sigma-Aldrich	100	MeOH	$C_{17}H_{22}ClNO$	255.16231	8.9	0.2; 3.6
Genistein		446-72-0	Extrasynthese	98	MeOH	$C_{15}H_{10}O_5$	270.05282	6.6; 8.1; 9.0	3.5; -2.4
				Surrogates					
Benzophenone-3-(phenyl-D5) [a]	$[^2H_5]$-BP3	1219798-54-5	Sigma-Aldrich	100	MeOH	$C_{14}D_5H_7O_3$	233.11000	7.1	3.6; 1.6
Benzyl Butyl Phthalate-D4 [a]	$[^2H_4]$-BnBuP	93951-88-3	Cambridge Isotope	98	MeOH	$C_{19}D_4H_{16}O_4$	316.16130	[b]	5.0; 5.0
Bisphenol-A-D16 [a]	$[^2H_{16}]$-BPA	96210-87-6	Sigma-Aldrich	98	MeOH	$C_{15}D_{16}O_2$	244.21540	9,8; 10,4	4.0; 3.5
Mono-(2-Ethyl-5-Oxohexyl) phthalate-^{13}C4 [a]	$[^{13}C_4]$-DEHP	1268238-35-2	Cambridge Isotope	99	MeOH	$^{13}C_4C_{12}H_{20}O_5$	292.13100	3.1	3.0; -0.3
				RTI compounds					
2-(4-chloro-2-methylphenoxy) acetic acid	MCPA	94-74-6	Sigma-Aldrich	98	MeOH	$C_9H_9ClO_3$	200.02402	3.4	2.4; -1.1
Abamectin		71751-41-2	Sigma-Aldrich	95	Acetone	$C_{48}H_{72}O_{14}$	872.49221	12.5	5.8; 5.8
Acephate		30560-19-1	Sigma-Aldrich	98	EtOH	$C_4H_{19}NO_3PS$	183.01190	10.5	-0.3; -0.6
Amitrole		61-82-5	Sigma-Aldrich	98	MeOH	$C_2H_4N_4$	84.04360	3.6; 11.1	-2.0; -1.6
Benodanil		15310-01-7	Sigma-Aldrich	98	MeOH	$C_{13}H_{10}INO$	322.98071	15.4	4.0; 4.0
Bromoxynil		1689-84-5	Sigma-Aldrich	98	MeOH	$C_7H_3Br_2NO$	274.85814	5.1	3.1; 1.0
Butocarboxim		34681-10-2	Sigma-Aldrich	98	MeOH	$C_7H_{14}N_2O_2S$	190.07760	1.3; 14.3	1.3; 1.3
Cefoperazone		113826-44-1	Sigma-Aldrich	98	Acetone	$C_{25}H_{27}N_9O_8S_2 \cdot 2H_2O$	645.14240	-2.0; 3.2	-0.2; -2.0

Table S1 – Continuation.

COMPOUND	Abbreviation	CAS number	Commercial vendor	Purity (%)	Solvent	Molecular formula	Exact mass (Da)	pKa	Log D (2.5; 10.5)
Chlormequat		999-81-5	Sigma-Aldrich	98	MeOH	$C_5H_{13}ClN$	122.07310	[b]	-3.3; -3.3
Coumaphos		56-72-4	Sigma-Aldrich	98	Acetone	$C_{14}H_{16}ClO_5PS$	362.01446	[b]	4.3; 4.3
Dichlorvos		62-73-7	Sigma-Aldrich	99	MeOH	$C_4H_7Cl_2O_4P$	219.94590	[b]	1.4; 1.4
Dinoterb		1420-07-1	Sigma-Aldrich	98	DMSO	$C_{10}H_{12}N_2O_5$	240.07462	5.2	3.1; 1.1
Inabenfide		82211-24-3	Sigma-Aldrich	98	Acetone	$C_{19}H_{15}ClN_2O_2$	338.08221	2.3; 13.5; 14.9	3.3; 3.5
Metamidophos		10265-92-6	Sigma-Aldrich	98	Isopropanol	$C_2H_8NO_2PS$	141.00134	15.6	-0.3; -0.3
Rifaximin		80621-81-4	Sigma-Aldrich	98	MeOH	$C_{43}H_{51}N_3O_{11}$	785.35236	5.9; 6.7	3.8; 1.1
Salinomycin		55721-31-8	Sigma-Aldrich	85	EtOH	$C_{42}H_{69}NaO_{11}\cdot x\ H_2O$	750.49181	4.5	7.5; 4.0
Simazine		122-34-9	Fluka	98	MeOH	$C_7H_{12}ClN_5$	201.07812	4.2; 14.8	0.2; 1.8
Tepraloxydim		149979-41-9	Sigma-Aldrich	95	MeOH	$C_{17}H_{24}ClNO_4$	341.13939	2.2	2.8; -0.8
Trichlorphon		52-68-6	Sigma-Aldrich	98.4	Isopropanol	$C_4H_8Cl_3O_4P$	255.92258	10.12	1.1; 0.6
Triclosan		3380-34-5	Sigma-Aldrich	99	MeOH	$C_{12}H_7Cl_3O_2$	287.95116	7.7	5.0; 2.9
Tylosin		1401-69-0	British Pharmacopoeia	100	MeOH	$C_{46}H_{77}NO_{17}$	915.51915	8.4; 12.5	-1.2; 2.3

[a]*Included in the target method,* [b]*Not ionizable atoms,* [c]*Only used in the enzymatic hydrolysis optimization.*

Table S2 - Mass spectrometry conditions for the target analysis of the 24 EDCs and 4 surrogates by LC-QqQ at pH 2.5 and 10.5, including the ionization mode, retention time (RT), *m/z* fragments, fragmentor voltage and collision energy (CE) for each compound.

Compound	pH 2.5						pH 10.5					
	Ionization mode	RT (min)	Precursor ion (*m/z*)	Product ion(s) (*m/z*)	Fragmentor voltage (V)	Collision-energy (eV)	Ionization mode	RT (min)	Precursor ion (*m/z*)	Product ion(s) (*m/z*)	Fragmentor voltage (V)	Collision-energy (eV)
						Benzophenones						
4OH-BP	$[M+H]^+$	7.0	199.1	121.1/105.1	105	17	$[M-H]^-$	2.6	197.1	92.0/120.0	75	33
BP1	$[M+H]^+$	8.7	215.1	137.0/81.1	135/120	17/41	$[M-H]^-$	2.8	213.1	135.0/91.0	135	17/29
BP2	$[M+H]^+$	5.2	247.1	137.0/81.1	120	21/41	$[M-H]^-$	0.7	245.0	135.0/109.0	105	13/21
BP3	$[M+H]^+$	11.2	229.0	151.0/105.0	75	21	$[M+H]^+$	11.0	229.0	151.0/105.0	75	21
BP8	$[M+H]^+$	9.9	245.1	121.1/151.1	105	13/17	$[M-H]^-$	6.4	243.1	123.0/93.0	120	13/21
						Bisphenols						
BPA	$[M+H]^+$	11.3	229.1	151.0/105.1	120	17	$[M-H]^-$	7.3	227.1	121.2/133.1	75	17/25
BPAF	$[M-H]^-$	10.2	335.0	265.1/245.1	150	21/37	$[M-H]^-$	7.1	335.0	265.1/197.0	120	21/41
BPAP	$[M-H]^-$	10.3	289.1	274.1/211.1	135	17/29	$[M-H]^-$	9.9	289.1	274.1/211.1	75	17/25
BPP	$[M-H]^-$	11.8	345.2	330.1/315.2	150	25/41	$[M-H]^-$	11.7	345.2	330.1/315.1	75	25/41
BPS	$[M+H]^+$	3.7	251.0	157.0/109.1	130	13/21	$[M-H]^-$	0.7	249.0	108.0/92.0	135	29/37
BPZ	[a]	[a]	[a]	[a]	[a]	[a]	$[M-H]^-$	10.4	267.1	173.1/223.1	75	29/33
BPA-G	[a]	[a]	[a]	[a]	[a]	[a]	$[M-H]^-$	3.7	403.2	113.1/227	75	17
						Parabens						
BnP	$[M-H]^-$	9.8	227.1	136.0/92.0	105	9/25	$[M-H]^-$	4.1	227.1	136.0/92.0	105	9/25
BuP	$[M+H]^+$	9.7	195.1	139.1/95.1	75	9/21	$[M-H]^-$	4.0	193.1	136.0/92.0	105	13/25
EDHB	$[M+H]^+$	4.3	183.1	155.1/111.1	75	9/13	[a]	[a]	[a]	[a]	[a]	[a]
EtP	$[M+H]^+$	5.7	167.1	139.1/95.1	75	9/17	$[M-H]^-$	1.9	165.1	137.0/92.0	90	13/25
MDHB	$[M+H]^+$	3.2	169.0	137.0/109.1	90	9/21	$[M-H]^-$	0.7	167.0	125.1/108.0	105	17
MeP	$[M+H]^+$	4.2	153.1	121.1/93.1	90	13/25	$[M-H]^-$	0.8	151.0	136.0/92.0	90	13/21

Table S2 – Continuation.

Compound	pH 2.5						pH 10.5					
	Ionization mode	RT (min)	Precursor ion (*m/z*)	Product ion(s) (*m/z*)	Fragmentor voltage (V)	Collision-energy (eV)	Ionization mode	RT (min)	Precursor ion (*m/z*)	Product ion(s) (*m/z*)	Fragmentor voltage (V)	Collision-energy (eV)
Phthalates												
MBnP	$[M+H]^+$	8.3	257.1	91.1/149.1	75	13/9	$[M+H]^+$	3.0	257.1	149.1/91.1	75	9/13
MBuP	$[M+H]^+$	8.0	223.1	149.0/121.0	75	9/13	[a]	[a]	[a]	[a]	[a]	[a]
MEHHP	$[M-H]^-$	8.6	293.1	121.0/145.1	105	17/9	[a]	[a]	[a]	[a]	[a]	[a]
MEHP	$[M+H]^+$	11.6	279.2	149.1/167.1	90	13/5	$[M+H]^+$	6.5	279.2	167.1/149.1	90	5/13
MEOHP	$[M-H]^-$	7.7	291.1	143.1/121	105	9/17	$[M-H]^-$	3.1	291.1	143.1/121.0	105	9/17
Antibacterials												
TCC	$[M-H]^-$	11.8	313.0	160.0/126.0	90	9/25	$[M-H]^-$	11.8	313.0	160.0/126.0	90	9/25
TCS	$[M-H]^-$	11.9	288.9/286.9[b]	35	90	13	$[M-H]^-$	9.5	288.9/286.9[b]	35	90	13
Surrogates												
$[^2H_5]$-BP3	$[M+H]^+$	11.2	234.1	151.0/82.0	120	13	$[M+H]^+$	11.0	234.1	151.0/110.0	105	17/21
$[^2H_{16}]$-BPA	$[M+H]^+$	11.4	243.1	215	120	17	$[M-H]^-$	7.3	241.0	223.0/151.0	135/105	17/33
$[^2H_4]$-BnBuP	$[M+H]^+$	12.0	317.4	209	75	10	$[M+H]^+$	12.0	317.4	209	75	10
$[^{13}C_4]$-DEHP	$[M-H]^-$ /$[M+H]^+$	7.6	295.00/297.20	124.0/281.1	90	9	[a]	[a]	[a]	[a]	[a]	[a]

[a]*Not ionized.*

Table S3 - Mass spectrometry conditions for the analysis by LC-qOrbitrap at pH 2.5 and 10.5 including the ionization mode, retention time (RT), precursor and product ions (*m/z*), as well as the instrumental limit of identification (iLOQ, ng g^{-1}) and the MS2 match for each compound.

COMPOUND	Abbreviation	MODE*	RT (min)	MS1 (m/z)	MS2 fragments (m/z)			iLOI	MS2 match (%)	
									Standard solution	Spiked urine
Benzophenones										
4,4'-bis(dimethylamino)benzophenone	4,4-DMA-BP	$[M+H]^+$	13.79	269.16484	148.07544	120.08074	105.05733	0.1	98	98
4-Hydroxybenzophenone[a]	4-OH-BP	$[M+H]^+$	10.36	199.07536	146.96149	128.95097	121.02863	1.4	98	98
		$[M-H]^-$	3.19	197.06080	116.92700	108.02055	95.01251	8.9	77	77
4-Methylbenzophenone	4-MeBP	$[M+H]^+$	13.69	197.09609	196.16920	119.04906	105.03356	1.0	86	86
4-Phenylbenzophenone	4-PhBP	$[M+H]^+$	15.44	259.11174	181.06433	105.03358	95.04932	1.0	92	[b]
Benzophenone-1[a]	BP1	$[M+H]^+$	11.71	215.07027	158.02715	141.00055	95.04950	5.3	76	76
		$[M-H]^-$	4.65	213.05572	169.06517	135.00787	91.01756	[c]	0	0
Benzophenone-2[a]	BP2	$[M+H]^+$	8.04	247.06010	137.02347	81.03397	69.07040	[c]	[b]	[b]
		$[M-H]^-$	0.65	245.04555	153.01894	135.00739	109.02801	9.5	98	98
Benzophenone-3[a]	BP3	$[M+H]^+$	14.03	229.08592	151.03893	105.03380	58.06586	2.6	98	98
		$[M-H]^-$	13.30	227.07137	186.89841	127.72902		7.8	75[d]	75[d]
Benzophenone-8[a]	BP8	$[M+H]^+$	12.54	245.08075	151.03914	121.02866	95.04950	10.8	98	[b]
		$[M-H]^-$	9.62	243.06628	123.04387	108.02020	93.03324	16.5	94	94
Bisphenols										
Bisphenol A[a]	BPA	$[M-H]^-$	9.79	227.10775	242.20034	154.96615		10.6	93	93
Bisphenol AF[a]	BPAF	$[M-H]^-$	10.19	335.05122	265.04822	177.03363	68.99443	8.0	94	94
Bisphenol AP[a]	BPAP	$[M-H]^-$	12.31	289.12415	274.09872	195.08142	93.03334	11.2	95	95
Bisphenol P[a]	BPP	$[M-H]^-$	14.65	345.18600	331.26770	313.25711	295.24600	[c]	0[d]	[b]
Bisphenol S[a]	BPS	$[M-H]^-$	0.65	249.02270	155.98839	108.02045	92.02539	5.1	90	0
Bisphenol Z[a]	BPZ	$[M-H]^-$	12.04	267.14087	223.07588	173.09627	145.06471	[c]	0[d]	[b]
Parabens										
Benzyl paraben[a]	BnP	$[M-H]^-$	13.33	227.07137	220.94827	136.01535	92.02540	10.0	67[d]	67[d]
Butyl paraben[a]	BuP	$[M+H]^+$	12.4	195.10157	167.07016			0.6	71[d]	71[d]
		$[M-H]^-$	7.4	193.08702	175.90752	136.01559	93.03306	22.2	90	90

Table S3 – Continuation.

COMPOUND	Abbreviation	MODE*	RT (min)	MS1 (m/z)	MS2 fragments (m/z)			iLOI	MS2 match (%)	
									Standard solution	Spiked urine
Ethyl 3,4-dihydroxybenzoate[a]	EDHB	$[M+H]^+$	8.3	183.06519	155.03375	111.04416		0.6	98	[b]
Ethyl paraben[a]	EtP	$[M+H]^+$	8.77	167.07027	139.03878	121.02843	84.96015	0.5	79[d]	100[d]
		$[M-H]^-$	1.74	165.05572	137.02332	95.01241	93.03318	21.9	91	91
Methyl 3,5-dihydroxybenzoate[a]	MDHB	$[M-H]^-$	0.74	167.03498	151.00580	108.02026	74.98932	10.7	75[d]	75[d]
Methyl paraben[a]	MeP	$[M+H]^+$	6.16	153.05462	121.02801	109.06575	94.04124	[c]	[b]	[b]
		$[M-H]^-$	1.09	151.04007	136.01511	95.01254	92.02537	8.4	82	82
Phthalates										
Mono-(2-ethyl-5-hydroxyhexyl) phthalate[a]	MEHHP	$[M+H]^+$	11.66	295.15400	277.21619	221.11699	149.02327	22.0	75[d]	75[d]
		$[M-H]^-$	6.89	293.13945	293.13996	276.97839	214.91333	153.0	67[d]	[b]
Mono-[(2RS)-2-Ethyl-5-oxohexyl phthalate[a]	MEOHP	$[M-H]^-$	5.97	291.12380	143.10657	121.02824	79.95606	146.0	100[d]	100[d]
Mono-2-Ethylhexyl phthalate[a]	MEHP	$[M-H]^-$	10.49	277.14453	226.30743	134.03613	121.02790	8.1	92	92
Mono-benzyl phthalate[a]	MBnP	$[M+H]^+$	11.52	257.08084	127.42988	91.05447	79.24430	10.7	50[d]	50[d]
		$[M-H]^-$	5.61	255.06628	183.08121	121.02831	107.04894	108.0	80[d]	80[d]
Mono-butyl phthalate[a]	MBuP	$[M-H]^-$	4.55	221.08193	134.03688	121.08283	71.04906	160.0	73	73
Antibacterials and biocides										
Triclocarban[a]	TCC	$[M-H]^-$	14.91	312.97077	159.97168	126.01044		5.8	84	[b]
Triclosan[a]	TCS	$[M-H]^-$	12.03	286.94389	160.95664	141.98271	89.79664	[c]	0	0
Acetamiprid		$[M+H]^+$	4.83	223.07450	187.09807	126.01057	56.05043	2.6	76	76
Alachlor		$[M+H]^+$	13.48	270.12553	238.09899	224.08363	148.11101	16.3	94	[b]
Ametryn		$[M+H]^+$	8.99	228.12774	198.10777	182.12785	164.11879	2.4	97	97
Atrazine		$[M+H]^+$	10.61	216.10105	174.05431	132.03244	104.00111	0.5	97	97
Boscalid		$[M+H]^+$	12.57	343.03994	307.06128	139.98996	112.03962	2.7	93	93
Chloridazon		$[M+H]^+$	4.83	222.04287	204.02152	104.04936	92.04980	1.0	92	92
Chloroxuron		$[M+H]^+$	13.08	291.08948	164.09488	72.04483	70.04045	0.5	67[d]	67[d]
Chlortoluron		$[M+H]^+$	10.3	213.07892	168.02075	91.54185	72.04487	[c]	42	42
Clomazone		$[M+H]^+$	11.82	240.07858	184.05276	125.01547	100.07620	1.0	92	92
Dichlorvos		$[M+H]^+$	8.32	220.95318	144.98145	127.01490	78.99486	6.0	89	89

Table S3 – Continuation.

COMPOUND	Abbreviation	MODE*	RT (min)	MS1 (m/z)	MS2 fragments (m/z)			iLOI	MS2 match (%)	
									Standard solution	Spiked urine
Diethyl Toluamide		$[M+H]^+$	10.87	192.13829	160.05139	119.04923	109.06506	0.5	97	97
Dimethachlor		$[M+H]^+$	11.48	256.10988	224.08359	148.11279		2.7	97	97
Dimethoate		$[M+H]^+$	4.63	230.00690	198.96478	170.96984	142.99290	16.4	95	95
Diuron		$[M+H]^+$	11.1	233.02429	187.96602	159.97048	72.04438	2.5	100	100
Ethion		$[M+H]^+$	15.71	384.99489	170.96992	142.93782	114.96184	1.0	70	[b]
Fenthion		$[M+H]^+$	14.33	279.02730	247.00217	169.01459	105.07076	5.4	50	[b]
Flusilazole		$[M+H]^+$	13.67	316.10761	316.10761	187.05935	165.07042	1.0	92	92
Imidacloprid		$[M+H]^+$	3.91	256.05958	209.05912	175.09810	84.05647	5.4	74	50
Linuron		$[M+H]^+$	12.19	249.01921	182.02292	159.97076	62.06075	5.5	97	97
Mecoprop		$[M-H]^-$	6.46	213.03240	141.00908	71.01210		[c]	36	36
Metazachlor		$[M+H]^+$	10.96	278.10547	210.06669	134.09567		2.5	95	95
Metconazole		$[M+H]^+$	14.36	320.15242	177.04610	125.01536	70.04045	1.1	96	96
Methiocarb		$[M+H]^+$	12.33	226.08963	190.12306	169.06891	121.06466	5.2	94	94
Metolachlor		$[M+H]^+$	13.53	284.14118	252.11448	176.14265		1.0	97	97
Metribuzin		$[M+H]^+$	8.21	215.09611	215.09611	187.10053	171.05918	5.6	95	42
Myclobutanil		$[M+H]^+$	12.88	289.12145	125.01538	81.07032	70.04094	1.0	98	98
Oryzalin		$[M+H]^+$	13.46	347.10198	305.05472	288.03045	243.01846	7.6	95	[b]
Pirimiphos-methyl		$[M+H]^+$	14.33	306.10358	278.07172	164.11891	136.08716	0.5	97	97
Prochloraz		$[M+H]^+$	13.81	376.03809	309.99634	265.95443	224.94491	2.4	94	94
Propachlor		$[M+H]^+$	10.94	212.08367	212.08367	170.03638	152.02598	2.4	88	88
Propanil		$[M+H]^+$	12.16	218.01340	161.98762	127.01882	57.03399	4.9	87	87
		$[M-H]^-$	12.12	215.99884	161.96835	159.97131		2.5	97	97
Propiconazole		$[M+H]^+$	14.2	342.07706	204.98201	186.97206	158.97547	1.0	97	97
Propyzamide		$[M+H]^+$	12.68	256.02905	256.02905	189.98132	172.95514	5.1	87	87
Pyrazophos		$[M+H]^+$	14.7	374.09340	346.06033	238.06414	222.08598	1.0	93	93
Quinmerac		$[M+H]^+$	5.06	222.03163	204.02204	176.02506	141.05754	5.6	97	[b]
Simazine		$[M+H]^+$	8.41	202.08540	132.03252	124.08717	96.05586	2.5	92	92

Table S3 – Continuation.

COMPOUND	Abbreviation	MODE*	RT (min)	MS1 (m/z)	MS2 fragments (m/z)			iLOI	MS2 match (%)	
									Standard solution	Spiked urine
Tebuconazole		$[M+H]^+$	14.01	308.15242	125.01546	70.04091		0.5	98	98
Terbuthylazine		$[M+H]^+$	12.37	230.11670	230.11670	174.05439	146.02335	0.5	98	98
Terbutryn		$[M+H]^+$	11.04	242.14339	186.08027	158.04968	91.03248	1.0	97	97
Thiacloprid		$[M+H]^+$	5.96	253.03092	186.01292	128.00648	126.01051	2.7	98	98
Triadimenol		$[M+H]^+$	13.06	296.11603	227.08246	99.08079	70.04094	5.4	99	99
Industrial chemicals										
2,4-Diethyl-9H-thioxanthen-9-one	DETX	$[M+H]^+$	16.82	269.09946	241.06763	213.03641		1.0	67[d]	[b]
2,4-Dinitrophenol		$[M-H]^-$	0.75	183.00377	123.00754	109.01551	95.01233	2.6	96	96
2-Hydroxybenzothiazole	2-OH-BTA	$[M+H]^+$	7.05	152.01646	124.02156	92.04994	80.05029	59.4	81	81
2-isopropylthioxanthone	ITX	$[M+H]^+$	16.08	255.08381	213.03667	184.03386		1.0	94	94
Benzothiazole		$[M+H]^+$	7.55	136.02155	109.01066	95.04934	65.03927	[c]	41	41
Caffeine		$[M+H]^+$	2.40	195.08765	138.06629	110.07108	83.06101	130.2	98	98
Cotinine		$[M+H]^+$	0.83	177.10224	98.06037	80.05003		[c]	42	42
PFAS										
Perfluorobutanesulfonic acid	PFBS	$[M-H]^-$	6.13	298.94299	154.84415	98.95438	79.95589	0.9	99	99
Perfluorooctanesulfonamide	PFOSA	$[M-H]^-$	11.65	497.94620	168.98850	77.96407		5.8	70	[b]
Perfluorooctanesulfonic acid	PFOS	$[M-H]^-$	11.71	498.93022	118.99095	98.95405	79.95600	1.0	96	96
Pharmaceuticals										
Acetaminophen		$[M+H]^+$	1.54	152.07061	135.11649	110.06015	93.03378	123.5	88	88
Diazepam		$[M+H]^+$	12.33	285.07892	222.11419	193.08941	154.04099	0.5	98	98
Diclofenac		$[M+H]^+$	13.96	296.02396	278.01364	250.01859	214.04192	2.4	98	98
Diphenhydramine		$[M+H]^+$	7.01	256.16959	167.08448	165.06853	152.06164	4.9	90	90
Genistein		$[M+H]^+$	9.30	271.06010	271.06010	215.06982	153.01763	5.5	99	99

**pH 2.5 for positive ionization mode and 10.5 for negative ionization mode. [a]Included in the target method, [b]No MS2 obtained, [c]Not calculated due to the absence of MS2 or MS2 match lower than 70 %, [d]in-silico fragmentation performed.*

Table S4 - Instrumental (iLOQ, ng g^{-1}) and procedural (pLOQ, ng g^{-1}) limits of quantification, upper limits (ng g^{-1}) and determination coefficients (r^2) of the calibration curves for LC-QqQ and LC-qOrbitrap at pH 2.5 and pH 10.5.

	LC-QqQ								LC-qOrbitrap							
	pH 2.5				pH 10.5				pH 2.5				pH 10.5			
Compounds	iLOQ	pLOQ	Upper limit	r^2	iLOQ	pLOQ	Upper limit	r^2	iLOQ	pLOQ	Upper limit	r^2	iLOQ	pLOQ	Upper limit	r^2
Benzophenones																
4OH-BP	0.3	0.07	180	0.9999	0.3	0.07	180	0.9998	1.4	0.3	180	0.9981	0.3	0.07	180	0.9999
BP1	0.5	0.1	155	0.9997	0.5	0.1	155	0.9999	2.6	0.7	155	0.9973	0.5	0.1	156	0.9999
BP2	0.2	0.2	144	0.9998	0.05	0.05	98	0.9996	4.9	4.7	144	0.9977	1.1	1.1	144	0.9994
BP3	0.5	0.5	157	0.9998	0.3	0.2	157	0.9999	1.2	1.1	157	0.9982	2.6	2.4	157	0.9995
BP8	0.3	0.1	165	0.9994	0.6	0.2	165	0.9999	1.3	0.5	165	0.9961	1.3	0.5	164	0.9994
Bisphenols																
BPA	0.3	0.08	161	0.9997	1.3	0.4	161	0.9996	[a]	[a]	[a]	[a]	0.5	0.2	161	0.9993
BPAF	0.3	0.1	111	0.9971	0.06	0.02	111	0.9968	[a]	[a]	[a]	[a]	2.7	1.1	111	0.9962
BPAP	11.3	5.7	117	0.9846	0.06	0.03	171	0.9999	[a]	[a]	[a]	[a]	1.4	0.7	171	0.9996
BPP	5.3	1.8	157	0.9937	0.3	0.09	107	0.9870	[a]	[a]	[a]	[a]	2.7	0.9	157	0.9931
BPS	0.3	0.2	103	0.9996	0.1	0.09	103	0.9997	[a]	[a]	[a]	[a]	0.5	0.4	152	0.9996
BPZ	[a]	[a]	[a]	[a]	1.3	0.6	163	0.9999	[a]	[a]	[a]	[a]	2.8	1.2	163	0.9954
Parabens																
BnP	0.1	0.06	152	0.9999	0.3	0.1	152	0.9998	[a]	[a]	[a]	[a]	2.6	1.3	152	0.9994
BuP	0.3	0.09	165	0.9999	0.6	0.2	165	0.9999	5.6	1.7	165	0.9890	0.3	0.09	165	0.9995
EDHB	1.3	0.3	112	0.9946	[a]	[a]	[a]	[a]	16.4	3.3	163	0.9891	[a]	[a]	[a]	[a]
EtP	0.6	0.2	111	0.9995	0.6	0.2	111	0.9989	10.7	3.1	163	0.9919	0.06	0.02	163	1.0000
MDHB	1.3	0.2	111	0.9948	0.6	0.08	111	0.9946	[a]	[a]	[a]	[a]	0.6	0.08	163	0.9997
MeP	0.6	0.1	171	0.9997	1.3	0.3	171	0.9994	11.2	2.3	171	0.9951	1.3	0.3	171	0.9990
Phthalates																
MBnP	0.06	0.02	160	0.9993	2.7	0.9	109	0.9802	2.7	0.9	160	0.9980	0.3	0.1	160	1.0000
MBuP	0.5	0.1	160	0.9992	[a]	[a]	[a]	[a]	[a]	[a]	[a]	[a]	0.5	0.1	160	0.9999
MEHHP	0.06	0.01	164	0.9999	[a]	[a]	[a]	[a]	8.1	1.8	164	0.9949	0.3	0.06	164	0.9999
MEHP	1.2	0.6	154	0.9945	2.6	1.2	105	0.9960	5.2	2.4	154	0.9975	0.3	0.1	105	0.9998

Table S4 – Continuation.

	LC-QqQ								LC-qOrbitrap							
	pH 2.5				pH 10.5				pH 2.5				pH 10.5			
Compounds	iLOQ	pLOQ	Upper limit	r^2	iLOQ	pLOQ	Upper limit	r^2	pLOQ	iLOQ	Upper limit	r^2	iLOQ	pLOQ	Upper limit	r^2
MEOHP	0.05	0.01	146	0.9999	2.5	0.5	146	0.9993	19.7	4.2	146	0.9905	1.2	0.2	146	0.9998
Antibacterials																
TCC	0.1	0.06	171	0.9996	0.1	0.06	116	0.9990	2.9	1.5	171	0.9968	1.4	0.7	171	0.9985
TCS	1.3	0.7	111	0.9983	0.3	0.2	163	0.9998	[a]	[a]	[a]	[a]	2.7	1.5	163	0.9983

[a]*Not ionized.*

Table S5 - Average (n=5) absolute (%) and apparent (%) recoveries and relative standard deviations (RSDs, %) obtained at the (a) low (3 ng g^{-1}), (b) medium (6 ng g^{-1}) and (c) high (30 ng g^{-1}) spiking levels for SPE-LC-QqQ at pH 2.5 and 10.5. Surrogate used for apparent recovery calculation is included when applicable.

(a)

	Day 1				Day 2				Day 3				
Compound	Absolute Recovery	RSD	Apparent Recovery	RSD	Absolute Recovery	RSD	Apparent Recovery	RSD	Absolute Recovery	RSD	Apparent Recovery	RSD	Surrogate
						Benzophenones							
4OH-BP	89	9	*a*	*a*	91	10	*a*	*a*	89	9	*a*	*a*	*a*
BP1	77	15	*a*	*a*	74	18	*a*	*a*	84	14	*a*	*a*	*a*
BP2	21	27	89	16	22	25	105	13	29	16	114	22	[$^{2}H_{5}$]-BP3
BP3	22	29	92	25	22	30	103	18	26	25	101	8	[$^{2}H_{5}$]-BP3
BP8	56	10	84	5	56	10	89	3	58	12	76	7	[$^{2}H_{16}$]-BPA
						Bisphenols							
BPA	71	27	106	25	70	25	112	22	75	27	98	25	[$^{2}H_{16}$]-BPA
BPAF	51	22	77	18	45	12	72	14	48	18	63	12	[$^{2}H_{16}$]-BPA
BPAP	40	22	60	18	38	22	61	19	45	25	59	18	[$^{2}H_{16}$]-BPA
BPP	*b*	*b*	*b*	*b*	*b*	*b*	*b*	*b*	*b*	*b*	*b*	*b*	*b*
BPS	24	20	101	12	22	19	103	18	29	7	113	7	[$^{2}H_{5}$]-BP3
BPZ	46	25	71	23	49	16	78	12	55	28	72	22	[$^{2}H_{16}$]-BPA
						Parabens							
BnP	39	24	61	20	38	10	51	8	40	17	52	15	[$^{2}H_{16}$]-BPA
BuP	64	15	96	13	68	5	108	5	58	10	76	10	[$^{2}H_{16}$]-BPA
EDHB	100	30	*a*	*a*	94	34	*a*	*a*	87	26	*a*	*a*	*a*
EtP	70	12	105	15	66	6	106	6	75	5	98	6	[$^{2}H_{16}$]-BPA
MDHB	133	54	87	18	139	43	95	14	128	27	85	9	[$^{13}C_{4}$]-DEHP
MeP	97	16	*a*	*a*	104	6	*a*	*a*	109	8	*a*	*a*	*a*

Table S5 – (a) Continuation.

Compound	Day 1 Absolute Recovery	RSD	Apparent Recovery	RSD	Day 2 Absolute Recovery	RSD	Apparent Recovery	RSD	Day 3 Absolute Recovery	RSD	Apparent Recovery	RSD	Surrogate
Phthalates													
MBnP	57	16	86	14	70	6	91	9	47	18	62	16	[$^{2}H_{16}$]-BPA
MBuP	112	16	75	19	161	20	111	21	120	29	79	25	[$^{13}C_{4}$]-DEHP
MEHHP	90	10	*a*	*a*	103	3	*a*	*a*	112	6	*a*	*a*	*a*
MEHP	44	35	101	28	47	27	103	24	60	24	114	22	[$^{2}H_{5}$]-BP3
MEOHP	93	11	*a*	*a*	111	7	*a*	*a*	118	7	*a*	*a*	*a*
Antibacterials													
TCC	*b*	*b*	*b*	*b*	*b*	*b*	*b*	*b*	*b*	*b*	*b*	*b*	*b*
TCS	*b*	*b*	*b*	*b*	*b*	*b*	*b*	*b*	*b*	*b*	*b*	*b*	*b*

[a]*Not calculated due to the absence of an appropriate surrogate,* [b]*Below instrumental limit of quantification.*

(b)

Compound	Day 1 Absolute Recovery	RSD	Apparent Recovery	RSD	Day 2 Absolute Recovery	RSD	Apparent Recovery	RSD	Day 3 Absolute Recovery	RSD	Apparent Recovery	RSD	Surrogate
Benzophenones													
4OH-BP	80	15	*a*	*a*	85	6	*a*	*a*	91	5	*a*	*a*	*a*
BP1	75	12	*a*	*a*	78	13	*a*	*a*	86	10	*a*	*a*	*a*
BP2	41	20	95	12	45	19	100	11	31	22	90	13	[$^{2}H_{5}$]-BP3
BP3	41	16	96	15	41	11	88	12	32	12	94	5	[$^{2}H_{5}$]-BP3
BP8	71	2	107	4	75	6	98	6	74	12	96	15	[$^{2}H_{16}$]-BPA
Bisphenols													
BPA	56	15	85	13	60	10	79	12	65	13	84	10	[$^{2}H_{16}$]-BPA
BPAF	65	14	98	13	69	15	89	12	63	10	82	8	[$^{2}H_{16}$]-BPA
BPAP	64	13	96	8	77	12	100	9	75	12	97	8	[$^{2}H_{16}$]-BPA
BPP	60	6	90	8	60	16	78	14	51	10	66	9	[$^{2}H_{16}$]-BPA
BPS	37	14	85	15	39	11	86	15	30	13	87	14	[$^{2}H_{5}$]-BP3

Table S5 – (b) Continuation.

	Day 1				Day 2				Day 3				
Compound	Absolute Recovery	RSD	Apparent Recovery	RSD	Absolute Recovery	RSD	Apparent Recovery	RSD	Absolute Recovery	RSD	Apparent Recovery	RSD	Surrogate
BPZ	61	13	92	10	73	15	96	16	74	14	96	12	[$^{2}H_{16}$]-BPA
Parabens													
BnP	57	18	85	16	59	15	77	10	55	14	71	12	[$^{2}H_{16}$]-BPA
BuP	73	10	109	11	74	4	96	6	64	5	83	7	[$^{2}H_{16}$]-BPA
EDHB	91	9	[a]	[a]	99	8	[a]	[a]	99	6	[a]	[a]	[a]
EtP	65	6	98	9	70	10	91	7	62	6	81	8	[$^{2}H_{16}$]-BPA
MDHB	104	3	[a]	[a]	104	24	[a]	[a]	103	11	[a]	[a]	[a]
MeP	93	2	[a]	[a]	89	10	[a]	[a]	98	6	[a]	[a]	[a]
Phthalates													
MBnP	74	4	104	8	80	8	104	12	80	4	118	10	[$^{2}H_{16}$]-BPA
MBuP	128	14	83	16	133	9	93	12	125	2	82	1	[$^{13}C_{4}$]-DEHP
MEHHP	94	2	[a]	[a]	96	7	[a]	[a]	104	8	[a]	[a]	[a]
MEHP	85	7	[a]	[a]	80	13	[a]	[a]	95	5	[a]	[a]	[a]
MEOHP	133	15	86	12	142	3	98	8	159	5	105	9	[$^{13}C_{4}$]-DEHP
Antibacterians													
TCC	38	10	87	16	44	15	98	21	30	13	87	21	[$^{2}H_{5}$]-BP3
TCS	36	12	81	9	33	14	73	14	29	16	84	20	[$^{2}H_{5}$]-BP3

[a]*Not calculated due to the absence of an appropriate surrogate*

(c)

	Day 1				Day 2				Day 3				
Compound	Absolute Recovery	RSD	Apparent Recovery	RSD	Absolute Recovery	RSD	Apparent Recovery	RSD	Absolute Recovery	RSD	Apparent Recovery	RSD	Surrogate
Benzophenones													
4OH-BP	90	9	[a]	[a]	88	5	[a]	[a]	86	3	[a]	[a]	[a]
BP1	78	17	[a]	[a]	86	8	[a]	[a]	84	8	[a]	[a]	[a]

Table S5 – (c) Continuation.

	Day 1				Day 2				Day 3				
Compound	Absolute Recovery	RSD	Apparent Recovery	RSD	Absolute Recovery	RSD	Apparent Recovery	RSD	Absolute Recovery	RSD	Apparent Recovery	RSD	Surrogate
BP2	34	20	105	17	46	4	84	15	33	7	114	21	[$^2H_5$]-BP3
BP3	35	24	109	13	31	17	102	11	28	11	97	3	[$^2H_5$]-BP3
BP8	67	15	94	16	77	11	100	8	71	7	107	7	[$^2H_{16}$]-BPA
Bisphenols													
BPA	56	21	78	24	60	3	77	6	64	6	96	6	[$^2H_{16}$]-BPA
BPAF	50	24	70	22	53	16	69	13	57	10	85	9	[$^2H_{16}$]-BPA
BPAP	59	27	82	23	67	19	87	16	64	18	96	16	[$^2H_{16}$]-BPA
BPP	42	12	59	13	43	21	56	18	49	19	74	17	[$^2H_{16}$]-BPA
BPS	32	18	110	17	34	13	112	14	35	10	122	8	[$^2H_5$]-BP3
BPZ	62	24	87	20	80	17	104	13	79	14	118	12	[$^2H_{16}$]-BPA
Parabens													
BnP	41	19	59	13	46	12	60	7	43	14	64	13	[$^2H_{16}$]-BPA
BuP	63	14	88	10	68	5	88	1	56	3	84	6	[$^2H_{16}$]-BPA
EDHB	95	8	[a]	[a]	81	10	[a]	[a]	82	13	[a]	[a]	[a]
EtP	71	13	103	11	75	4	93	3	73	11	93	13	[$^2H_{16}$]-BPA
MDHB	103	16	[a]	[a]	107	6	[a]	[a]	104	8	[a]	[a]	[a]
MeP	109	13	[a]	[a]	115	5	[a]	[a]	115	4	[a]	[a]	[a]
Phthalates													
MBnP	64	18	96	15	72	6	115	8	86	15	112	13	[$^2H_{16}$]-BPA
MBuP	81	17	[a]	[a]	76	7	[a]	[a]	91	7	[a]	[a]	[a]
MEHHP	99	8	[a]	[a]	114	5	[a]	[a]	137	9	[a]	[a]	[a]
MEHP	81	22	[a]	[a]	88	8	[a]	[a]	104	15	[a]	[a]	[a]
MEOHP	100	18	[a]	[a]	114	7	[a]	[a]	136	18	[a]	[a]	[a]
Antibacterials													
TCC	29	19	89	24	22	25	72	25	22	19	77	22	[$^2H_5$]-BP3
TCS	26	22	82	26	27	34	89	24	24	32	84	25	[$^2H_5$]-BP3

[a]*Not calculated due to the absence of an appropriate surrogate.*

Table S6 – Compounds annotated at the volunteers´ samples at levels 1-5 using suspect and non-target screening by SPE-LC-qOrbitrap.

Feature	Exact mass	Exact mass error (ppm)	Formula	Name	RT (min)	Standard RT/Predicted RT	Confidence level	Use/Application	Volunteers			
									A	B	C	D
Suspect screening												
1	151.06319	-0.90	C8 H9 N O2	Acetaminophen	1.44	1.54	1	Pharmaceutical	✓	✓	✓	✓
2	176.09482	-0.81	C10 H12 N2 O	Cotinine	0.92	0.83	1	Metabolite of nicotine	✓	✓	✓	X
3	194.08020	-0.93	C8 H10 N4 O2	Caffeine	2.37	2.40	1	Psychostimulant drug	✓	✓	✓	✓
4	222.08870	-2.15	C12 H14 O4	MBuP	4.61	4.55	1	Plasticizer	✓	✓	✓	✓
5	228.07860	-0.25	C14 H13 O3	BP3	13.21	13.30	1	UV filter	✓	✓	✓	✓
6	228.11466	-1.63	C15 H16 O2	BPA	9.76	9.79	1	Plasticizer	✓	✓	X	X
7	254.05752	-1.52	C15 H10 O4	Daidzein	7.08	7.05	1	Food natural ingredient	✓	✓	✓	✓
8	270.05238	-1.64	C15 H10 O5	Genistein	8.44	9.30	1	Food natural ingredient<br>Personal Care Product	✓	✓	✓	✓
9	278.15130	-1.78	C16 H22 O4	MEHP	10.59	13.74	1	Plasticizer	✓	✓	✓	✓
10	99.06859	1.77	C5 H9 N O	N-Methyl-2-pyrrolidone	1.37	3.10	2a	Household & Commercial Products	✓	X	✓	✓
11	110.07326	0.88	C7 H10 O	(2E,4E)-Hepta-2,4-dienal	11.41	8.10	2a	Food additive (Flavouring agent)	X	X	X	✓
12	133.05268	-0.64	C8 H7 N O	4-Hydroxyindole	1.62	4.06	2a	Cosmetics (Hair dyeing)	X	✓	✓	✓
13	143.07341	-0.62	C10 H9 N	6-Methylquinoline	4.85	7.01	2a	Food natural ingredient<br>Food additive (Flavouring agent)	✓	✓	✓	✓
14	145.05265	-0.80	C9 H7 N O	1H-Indole-3-carboxaldehyde	4.11	5.93	2a	Food natural ingredient<br>Food additive (Flavouring agent)	✓	✓	✓	✓
15	147.06834	-0.51	C9 H9 N O	1-Furfurylpyrrole	5.74	4.77	2a	Food additive (Flavouring agent)	X	✓	✓	✓
16	168.04211	-0.86	C8 H8 O4	3,4-Dihydroxyphenylacetic acid	2.50	4.84	2a	Drug product	✓	✓	✓	✓
17	320.23521	0.21	C20 H32 O3	Tridecyl salicylate	12.57	14.86	2a	Cosmetics (Antistatic; Skin conditioning)	✓	✓	X	X
18	120.05756	0.36	C8 H8 O	Benzaldehyde, 3-methyl-	12.45	11.49	2b	Food natural ingredient<br>Food additive (Flavouring agent)	✓	✓	✓	X
19	135.06831	-0.75	C8 H9 N O	Acetanilide	2.33	5.67	2b	Cosmetics (Stabilizing)	X	✓	X	X
20	154.02654	-0.43	C7 H6 O4	2,6-Dihydroxybenzoic acid	5.86	6.47	2b	Synthetic Polymer	✓	✓	✓	X

Table S6 – Continuation.

Feature	Exact mass	Exact mass error (ppm)	Formula	Name	RT (min)	Standard RT/Predicted RT	Confidence level	Use/Application	Volunteers			
									A	B	C	D
**21**	236.14090	-1.47	C14 H20 O3	Benzene, 1-(1-ethoxyethoxy)-2-methoxy-4-(1-propen-1-yl)-	13.59	11.62	2b	Chemical substance	✓	✓	✓	✓
**22**	166.11048	-0.80	C9 H14 N2 O	2-Isobutyl-3-methoxypyrazine	5.45	5.17	3	Food natural ingredient<br>Food additive (Flavouring agent)	✓	✓	✓	✓
				3-sec-Butyl-2-methoxypyrazine		6.02		Food natural ingredient<br>Food additive (Flavouring agent)	✓	✓	✓	✓
**23**	180.06453	-1.08	C7 H8 N4 O2	Theobromine	1.40	2.55	3	Food (stimulant)	✓	✓	✓	✓
				Theophylline		2.50		Food (stimulant)<br>Pharmaceutical	✓	✓	✓	✓
**24**	180.06456	-0.91	C7 H8 N4 O2	Theobromine	1.67	2.55	3	Food (stimulant)	✓	✓	✓	✓
				Theophylline		2.50		Food (stimulant)<br>Pharmaceutical	✓	✓	✓	✓
**25**	180.07844	-1.12	C10 H12 O3	Propylparaben	10.29	7.39	3	Food additive (Preservative)	✓	✓	✓	✓
				Isopropylparaben		7.58		Food additive (Preservative)	✓	✓	✓	✓
				Benzoic acid, 4-methoxy-, ethyl ester		8.04		Food additive (Preservative)	✓	✓	✓	✓
**26**	180.07845	-1.06	C10 H12 O3	1,2-Ethanediol, 1-phenyl-, 2-acetate	5.20	5.91	3	Chemical substance	✓	✓	✓	✓
				Ethyl o-anisate		7.49		Food additive (Flavouring agent)				
				Benzoic acid, 4-methoxy-, ethyl ester		8.04		Food additive (Flavouring agent)				
				Propylparaben		7.39		Food additive (Preservative)				
				Isopropylparaben		7.58		Food additive (Preservative)				
				4-Methoxybenzyl acetate		5.97		Food additive (Flavouring agent)				
				Propylparaben		7.39		Food additive (Preservative)				
**27**	180.07847	-0.98	C10 H12 O3	Ethyl o-anisate	7.83	7.49	3	Food additive (Flavouring agent)	✓	✓	✓	✓
			C10 H12 O3	Benzoic acid, 4-methoxy-, ethyl ester		8.04		Food additive (Flavouring agent)				
			C10 H12 O3	4-Methoxybenzyl acetate		5.97		Food additive (Food improvement)				
			C10 H12 O3	Propylparaben		7.39		Food additive (Preservative)				
			C10 H12 O3	Isopropylparaben		7.58		Food additive (Preservative)				
			C10 H12 O3	Propylparaben		7.39		Food additive (Preservative)				

Table S6 – Continuation.

Feature	Exact mass	Exact mass error (ppm)	Formula	Name	RT (min)	Standard RT/Predicted RT	Confidence level	Use/Application	Volunteers			
									A	B	C	D
28	182.04381	-1.01	C6 H6 N4 O3	1-Methyluric acid	1.12	0.95	3	Metabolite of theophylline	✓	✓	✓	X
				4,9-Dihydro-3-methyl-1H-purine-2,6,8(3H)-trione		1.34		A methyl derivative of uric acid				
				7,9-Dihydro-7-methyl-1H-purine-2,6,8(3H)-trione		0.99		Metabolite of caffeine				
29	226.15660	-1.30	C13 H22 O3	Methyl 2-hexyl-3-oxocyclopentanecarboxylate	9.13	12.28	3	Cosmetics (Fragrance)	✓	✓	✓	✓
				Methyl dihydrojasmonate		11.84		Food additive (Flavouring agent)				
				1-Oxaspiro[2.5]octane-2-carboxylic acid, 5,5,7-trimethyl-, ethyl ester		11.73		Cosmetics (Fragrance)				
30	228.07859	-0.25	C14 H12 O3	Benzyl salicylate	7.77	11.04	3	Cosmetics (Fragrance, UV filter)	✓	✓	✓	✓
				Benzyl 4-hydroxybenzoate		10.15		Cosmetics (Antimicrobial)				
31	270.05238	-1.64	C15 H10 O5	4',6,7-Trihydroxyisoflavone	8.44	8.15	3	Food natural ingredient	✓	✓	✓	✓
				Apigenin		9.45		Food natural ingredient Pharmaceutical				
32	272.06807	-1.50	C15 H12 O5	Naringenin	7.92	8.90	3	Food natural ingredient Pharmaceutical	✓	✓	✓	✓
				Dihydrogenistein		8.38		Metabolite of genistin				
33	278.15122	-2.13	C16 H22 O4	Dibutyl phthalate	10.79	13.28	3	Personal Care Product, Plasticizer	✓	✓	✓	✓
				Dibutyl phthalate		13.28		Personal Care Product, Plasticizer				
				Diisobutyl phthalate		12.50		Plasticizer				
				Dibutyl phthalate		13.28		Personal Care Product, Plasticizer				
				Dibutyl phthalate		13.28		Personal Care Product, Plasticizer				
				Diisobutyl phthalate		12.50		Plasticizer				
34	109.05285	0.77	C6 H7 N O		1.44		4		X	X	✓	X
35	109.05290	1.29	C6 H7 N O		0.78		4		✓	✓	✓	X
36	126.03169	0.00	C6 H6 O3		1.87		4		✓	✓	✓	✓
37	129.05780	-0.35	C9 H7 N		6.49		4		✓	✓	✓	✓
38	129.05781	-0.34	C9 H7 N		7.57		4		✓	✓	✓	✓

Table S6 – Continuation.

Feature	Exact mass	Exact mass error (ppm)	Formula	Name	RT (min)	Standard RT/Predicted RT	Confidence level	Use/Application	Volunteers			
									A	B	C	D
39	134.07310	-0.48	C9 H10 O		5.20		4		✓	✓	✓	✓
40	134.07313	-0.28	C9 H10 O		9.38		4		✓	X	✓	X
41	136.05231	-0.87	C8 H8 O2		4.68		4		✓	✓	✓	✓
42	137.04761	-0.52	C7 H7 N O2		3.02		4		✓	✓	✓	✓
43	150.06797	-0.70	C9 H10 O2		9.90		4		✓	✓	✓	✓
44	152.11994	-1.16	C10 H16 O		7.36		4		✓	✓	✓	✓
45	152.11994	-1.13	C10 H16 O		8.36		4		✓	✓	✓	✓
46	152.11999	-0.85	C10 H16 O		6.27		4		✓	✓	✓	✓
47	152.12000	-0.77	C10 H16 O		9.03		4		✓	✓	✓	✓
48	194.05775	-0.84	C10 H10 O4		3.93		4		✓	✓	✓	✓
49	218.16678	-1.32	C15 H22 O		8.91		4		✓	✓	✓	✓
50	218.16678	-1.30	C15 H22 O		12.97		4		X	X	✓	X
51	218.16679	-1.27	C15 H22 O		11.10		4		X	✓	✓	✓
52	228.07836	-1.25	C14 H12 O3		13.21		4		✓	✓	X	X
53	242.09395	-1.43	C15 H14 O3		7.92		4		✓	✓	✓	X
54	117.05791	0.48			1.77		5		X	✓	✓	✓
55	122.03680	0.19			1.72		5		✓	✓	✓	✓
56	122.03681	0.25			2.50		5		✓	✓	✓	✓
57	131.07342	-0.57			1.57		5		X	✓	✓	✓
58	134.07314	-0.21			13.53		5		✓	X	X	X
59	136.05233	-0.76			1.75		5		✓	✓	✓	✓
60	137.04761	-0.50			11.96		5		✓	✓	✓	✓
61	138.03160	-0.70			2.13		5		✓	✓	✓	✓
62	143.07341	-0.59			1.81		5		✓	✓	✓	X
63	150.06799	-0.60			12.05		5		✓	✓	✓	✓
64	180.07844	-1.12			10.29		5		✓	✓	✓	✓
65	252.06314	-0.97			2.03		5		✓	✓	✓	✓
66	254.05789	-0.07			0.94		5		✓	X	✓	✓

Table S6 – Continuation.

Feature	Exact mass	Exact mass error (ppm)	Formula	Name	RT (min)	Standard RT/Predicted RT	Confidence level	Use/Application	Volunteers			
									A	B	C	D
				Non-target screening								
1	274.12368	-1.70	C16 H19 Cl N2	Chlorpheniramine	4.21	6.14	2a	Pharmaceutical (Antipuritic drug)	✓	✓	✓	✓
2	244.05178	-1.40	C9 H12 N2 O4 S	Pidotimod	2.05	3.01	2b	Pharmaceutical (Inmunomudulator)	X	X	✓	X
3	312.07799	-1.70	C13 H16 N2 O5 S	Epithienamycin B	1.91	3.91	2b	Pharmaceutical (Antibiotic)	X	X	✓	X
4	312.11438	-1.98	C14 H20 N2 O4 S	PS-6 / Penicillin F isomers	5.11	6.83	3	Pharmaceutical (Anbitiotic)	X	X	✓	X
5	217.07726	-0.75			6.71		5		✓	X	X	X
6	232.11332	-0.85			8.42		5		✓	✓	✓	✓
7	244.05178	-1.05			1.65		5		✓	✓	✓	✓
8	261.05566	-1.45			7.70		5		X	X	✓	X
9	329.09331	-1.75			3.16		5		X	X	✓	X

REFERENCES

[1] B. A. Rocha *et al.*, ‘A fast method for bisphenol A and six analogues (S, F, Z, P, AF, AP) determination in urine samples based on dispersive liquid-liquid microextraction and liquid chromatography-tandem mass spectrometry’, *Talanta*, vol. 154, pp. 511–519, Jul. 2016, doi: 10.1016/j.talanta.2016.03.098.

[2] O. González, M. E. Blanco, G. Iriarte, L. Bartolomé, M. I. Maguregui, and R. M. Alonso, ‘Bioanalytical chromatographic method validation according to current regulations, with a special focus on the non-well defined parameters limit of quantification, robustness and matrix effect’, *J. Chromatogr. A*, vol. 1353, pp. 10–27, Aug. 2014, doi: 10.1016/j.chroma.2014.03.077.

[3] R. Aalizadeh *et al.*, ‘Development and Application of Liquid Chromatographic Retention Time Indices in HRMS-Based Suspect and Nontarget Screening’, *Anal. Chem.*, Aug. 2021, doi: 10.1021/acs.analchem.1c02348.

[4] E. L. Schymanski *et al.*, ‘Identifying Small Molecules via High Resolution Mass Spectrometry: Communicating Confidence’, *Environ. Sci. Technol.*, vol. 48, no. 4, pp. 2097–2098, Feb. 2014, doi: 10.1021/es5002105.
